# Liuwei Dihuang pills ameliorate renal injury in experimental type 2 diabetes mellitus rat by regulating host-gut microbiota interaction

**DOI:** 10.3389/fphar.2025.1715600

**Published:** 2026-01-09

**Authors:** Han Jiang, Xiao-wan Hu, Xu Deng, Xian-jie Huang, Yu-lin Chen, Yi-fan Yang, Yan Du, Shuai Ji, Dao-quan Tang

**Affiliations:** 1 Jiangsu Key Laboratory of New Drug Research and Clinical Pharmacy, Xuzhou Medical University, Xuzhou, China; 2 Department of Pharmacy, Suining People’s Hospital Affiliated to Xuzhou Medical University, Suining, China; 3 Department of Pharmacy, Xuzhou Central Hospital, Xuzhou Clinical School of Xuzhou Medical University, Xuzhou, Jiangsu, China; 4 Department of Pharmaceutical Analysis, Xuzhou Medical University, Xuzhou, China

**Keywords:** diabetic kidney disease, gut microbiota, Liuwei Dihuang pills, metabolism disorder, renal impairment, TGF-β/Smad signaling pathway

## Abstract

**Background:**

Liuwei Dihuang pills (LW) are widely used as the traditional tonic prescription for the treatment of diabetes and diabetic kidney disease (DKD). This study aimed to investigate the potential mechanism underlying LW-mediated prevention and treatment of DKD from the perspective of host-gut microbiome co-metabolism.

**Methods:**

A rat model of DKD was established using high-fat diet and streptozotocin. Levels of type IV collagen (Col IV), fibronectin (FN), laminin (Lam), transforming growth factor-β (TGF-β), SMAD family member 7 (SMAD7), and SMAD3 in the kidneys were determined by real time-polymerase chain reaction and Western blot. Fecal metabolites were profiled using ultra-high-performance liquid chromatography-tandem mass spectrometry. Metagenomic sequencing of the feces was performed using high-throughput sequencing.

**Results:**

When combined with metformin (MET)-based therapy, LW significantly improved serum creatinine and blood urea nitrogen levels, kidney index, 24-h urine volume, urine protein content and excretion rate, and urinary creatinine and cystatin C levels. It also attenuated morphological changes. Correspondingly, LW intervention reduced the renal expression of TGF-β, SMAD3, Col IV, LAM, FN, interleukin (IL)-6, and IL-1β, while increasing SMAD7 expression. Additionally, it normalized metabolic pathway abnormalities in galactose, butyric acid, fructose, mannose, amino sugar, and nucleotide sugar metabolism. Moreover, LW regulated bacterial imbalances, notably in specific species such as *Allobaculum unclassified*, *Escherichia coli*, *Pseudoflavonifractor capillosus*, *Desulfovibrio porci*, *Oscillibacter* sp. *CU971*, *Parablautia muri*, *Phocaeicola dorei*, *Phocaeicola faecalis*, *Phocaeicola vulgatus*, and *Raoultella unclassified*.

**Conclusion:**

The combination of LW and MET ameliorated renal impairment in DKD rats by regulating the TGF-β/SMAD signaling pathway, metabolic disturbances in endogenous metabolites, and gut microbiota dysbiosis.

## 1 Introduction

With socioeconomic advancements, diabetes mellitus (DM) has emerged as a critical public health challenge. Projections indicate that the global diabetic population will reach 643 million by 2030, with type 2 diabetes mellitus (T2DM) accounting for 90% of the cases. T2DM is a metabolic disorder characterized by progressive decline in insulin function. At advanced stages, it induces diabetic renal injury, known as diabetic kidney disease (DKD). As a microvascular complication of diabetes, DKD is one of the primary etiologies of end-stage renal disease (ESRD), manifesting considerable morbidity and mortality burdens (Thipsawat, 2021). The hallmark clinical manifestations of DKD include decreased glomerular filtration rate (GFR), elevated urinary albumin excretion (≥300 mg/day), and progression to end-stage renal failure (Dagar et al., 2021). The pathogenesis of DKD is multifactorial, with persistent hyperglycemia-induced metabolic derangements serving as the primary drivers. The contributing factors include hemodynamic alterations, oxidative stress, and genetic predispositions. Stringent glycemic and blood pressure control has been empirically demonstrated to incompletely halt progression to ESRD or reduce DKD-related mortality (Samsu, 2021). Consequently, elucidating the underlying pathogenic mechanisms and pharmacological interventions for DKD are essential for developing effective therapeutic strategies.

Disrupted metabolic pathways involving amino acids (AAs), nucleotides, carbohydrates, and lipids substantially contribute to the pathogenesis and progression of DKD (Pereira et al., 2022). Accumulating evidence indicates that renal function modulates amino acids (AAs) metabolic homeostasis, whereas perturbations in AAs homeostasis reciprocally impair renal-dominated AAs metabolism (Liu L. et al., 2022). For instance, enhanced taurine, hypotaurine, tryptophan, and tyrosine metabolism ameliorates renal function and preserves kidney morphology (Li et al., 2022); elevated branched-chain amino acids (BCAAs) potentiate mammalian target of rapamycin (mTOR) signaling, accelerating renal tubular epithelial-mesenchymal transition (EMT) (Deng et al., 2025), and hyperglycemia-driven fatty acid (FAs) synthesis and triglyceride (TG) accumulation induce aberrant lipid metabolism, thereby increasing the risk of renal deterioration and cardiovascular mortality in T2DM (Herman-edelstein et al., 2014). Moreover, adipose accumulation from FAs synthesis triggers insulin resistance (IR). IR coupled with dysregulated lipid metabolism critically underpins the onset and advancement (Ormazabal et al., 2018). Hyperglycemia-induced oxidative stress further induces DNA damage and nucleotide metabolism impairments. Elevated urinary adenine observed in renal failure patients with albuminuria and diabetic individuals at high all-cause mortality risk suggests the potential of adenine as an early biomarker for renal injury (Sharma et al., 2023).

The gut microbiota exhibited dysbiosis in DKD. Preclinical studies revealed that DKD murine models displayed a significantly increased *Firmicutes/Bacteroidetes* ratio, with *Heterobacteria* and B*acillus anaerobicus* genera correlating with reduced GFR. Conversely, *Blautia* spp. may serve as a protective microbial taxon. In severe proteinuria models, *Allobaculum* and *Anaerosporobacter* genera were enriched, whereas mice with microalbuminuria demonstrated higher *Blautia* abundance than healthy controls (Li et al., 2020). Clinical investigations have indicated that *Firmicutes* phylum predominates in DKD patients' guts, followed by *Bacteroides*, *Clostridium*, and *Proteus*. In subjects without DKD, *Bacteroides* constituted the primary phylum, followed by *Firmicutes*, *Proteus*, and *Clostridium* (Chen R. et al., 2022). Notably, the gut microbiota in healthy individuals showed the highest *Prevotella* abundance, whereas *Bacteroides* emerged as the predominant genus in patients with T2DM and DKD, suggesting that shifts in dominant flora critically contribute to DKD progression (Zhang et al., 2022). Moreover, a valuable pre-clinical and clinical study showed that the genus *Lactobacillus* and the species *Lactobacillus johnsonii* were positively correlated with renal function decline in rats and patients with chronic kidney disease (CKD) (Miao et al., 2024a; Miao et al., 2024b).

The pathogenesis of DKD involves synergistic contributions from compromised host metabolic functions and gut microbiota dysbiosis, collectively driving aberrant endogenous metabolite profiles. Previous studies have shown that the reduced abundance of short-chain fatty acid (SCFAs)-producing bacteria such as Ruminococcaceae, Lachnospiraceae, and Bacteroidaceae in DKD patients is closely correlated with depleted acetate levels, which are critical modulators of energy metabolism, inflammatory pathways, glucose/lipid homeostasis, and insulin sensitivity (Chen T. et al., 2022). Gram-negative bacteria-derived lipopolysaccharides (LPS) can bind to toll-like receptor 4 (TLR4), activating the nuclear factor-kappa B (NF-κB) signaling pathway via reactive oxygen species (ROS) generation. This cascade promotes metabolic endotoxemia, low-grade chronic inflammation, increased adiposity, and IR (Boutagy et al., 2016). Overexpression of renal solute carrier organic anion transporter family member 4C1 (SLCO4C1) elevates gut-derived phenyl sulfate, inducing intrarenal accumulation that exacerbates proteinuria (Kikuchi et al., 2019). Concurrently, the composite index formed by the plasma levels of four uremic solutes, namely p-cresyl sulfate, indoxyl sulfate, hippurate, and phenylacetylglutamine, is positively associated with elevated creatinine and urea levels and could serve as a biomarker of renal failure (Shafi et al., 2015).

Recently, the protective effects of traditional Chinese medicine (TCM) in CKD or DKD have garnered significant attention from researchers worldwide (Li et al., 2023; Zheng et al., 2023). Numerous studies have demonstrated that TCM and its active components exert kidney-protective effects by modulating oxidative stress, inflammation, TGF-β/Smad signaling pathways, and gut microbiota dysbiosis (Feng et al., 2025; Liu et al., 2025; Miao et al., 2025). Liuwei Dihuang pills (LW), a classical kidney yin-nourishing formula formulated by Qian Yi during the Song Dynasty, consist of six medicinal components: Rehmanniae Radix Praeparata (Shu Di Huang, the prepared radix of *Rehmannia glutinosa* Libosch.), Dioscoreae Rhizoma (Shan Yao, Rhizoma of *Dioscorea japonica* Thunb.), Corni Fructus (Shan Zhu Yu, Fructus of *Cornus officinalis* Sieb. et Zucc.), Poria (Fu Ling, Sclerotium of *Poria cocos* (Schw.) Wolf), Moutan Cortex (Mu Dan Pi, Cortex of *Paeonia suffruticosa* Andrews), and Alismatis rhizoma (Ze Xie, Rhizoma of *Alisma orientale* Juzep.). In contemporary practice, LW monotherapy or in combination with conventional pharmaceuticals has emerged as a widely utilized complementary approach for managing or preventing DKD in China (Lin et al., 2016). Previous mechanistic studies have demonstrated that LW exerts renal protection effects through multimodal signaling regulation. For example, LW enhances cellular autophagy and ameliorates podocyte injury by modulating the phosphatidylinositol-3-kinase (PI3K)/protein kinase B (Akt)/mTOR axis, significantly reducing serum creatinine, urea nitrogen, and 24-h urinary protein excretion (Zhu et al., 2022). This formula can upregulate cytoglobin expression while suppressing pro-fibrotic pathways, including transforming growth factor-β (TGF-β)/SMADs, mitogen-activated protein kinases (MAPK), and NF-κB, which attenuate renal fibrosis and glomerulosclerosis (Xu et al., 2017).

Despite studies demonstrating that LW ameliorates cognitive dysfunction in aging mice through the gut microbiome or metabolome modulation (Liu B. et al., 2022), research on LW for DKD prevention remains understudied within these omics frameworks. Crucially, no investigation has elucidated LW’s reno-protective mechanisms of LW from a host-gut microbiota co-metabolism perspective. Synthesizing existing evidence, we propose that host-microbial co-metabolism drives DKD pathogenesis, whereas LW concurrently corrects host metabolic dysregulation and gut microbial dysbiosis to exert therapeutic effects. To validate this hypothesis, the current study employed streptozotocin (STZ)-induced T2DM rats maintained on a high-fat diet as research subjects to investigate the effects of LW in combination with metformin (MET) on biochemical indicators and renal tissue pathology. Additionally, pathway alterations underlying the attenuation of DKD progression, metagenomic gut microbiota, and metabolomic analyses of feces will be utilized to elucidate the potential mechanisms underlying the prevention and treatment of DKD.

## 2 Methods

### 2.1 Materials and reagents

**FIGURE 1:**
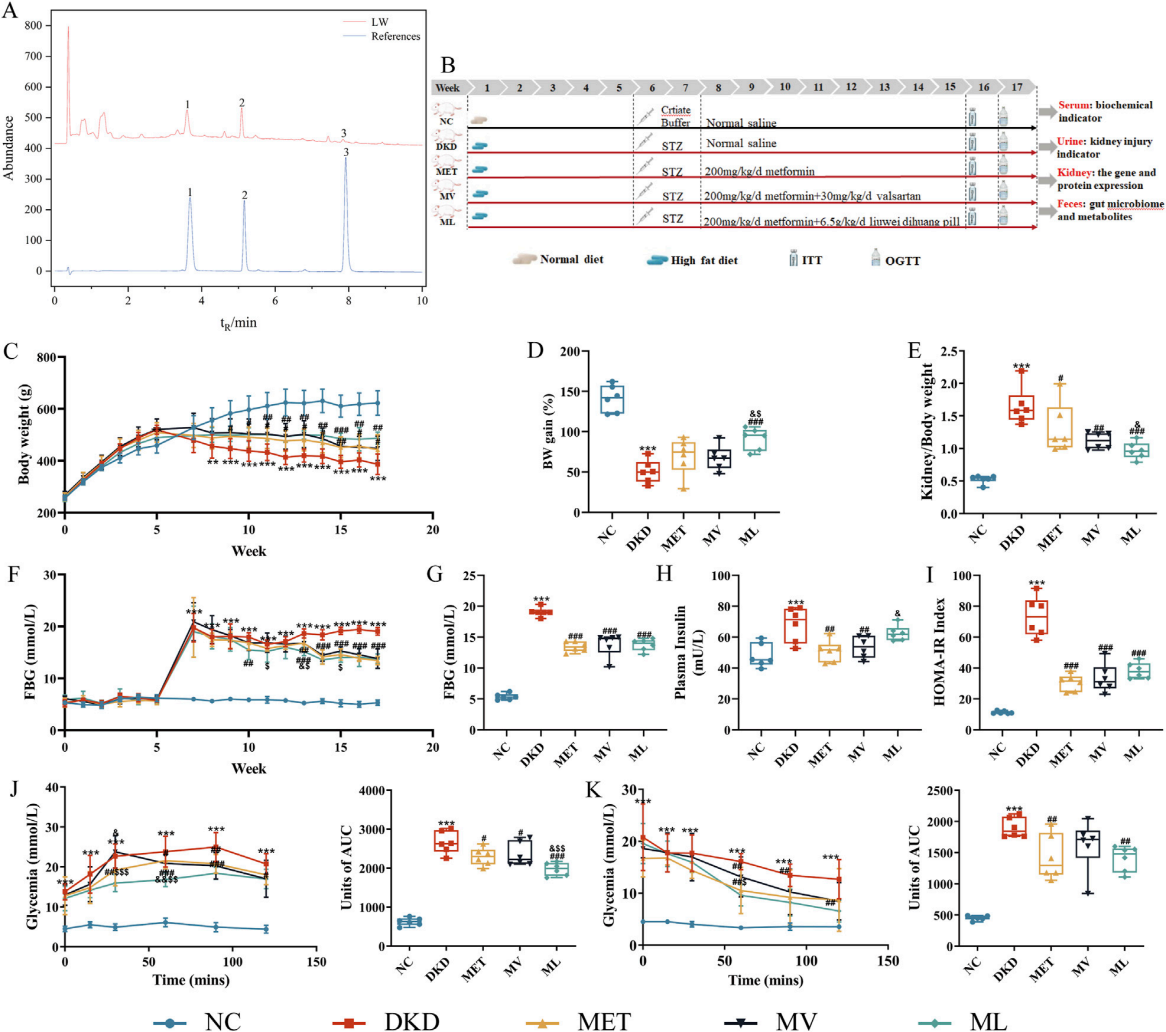
**(A)** Representative ultra-high performance liquid chromatography (UPLC) coupled with diode array detector (DAD) chromatogram of the standard references (Down, blue) and Liuwei Dihuang pill (LW) (Uo, black) (1. Loganin, 2. Morroniside, 3. Paeonol), **(B)** Workflow of animal experiments, **(C)** Body weight (BW) changes, **(D)** BW gain, **(E)** Kidney index, **(F)** Fasting blood glucose (FBG) changes, **(G)** FBG at week 17, **(H)** Fasting plasma insulin, **(I)** Homeostasis model assessment of insulin resistance (IR) (HOMA- IR), **(J)** Area under the curve (AUC) for an oral glucose tolerance test (OGTT) and Units of AUC for OGTT, **(K)** AUC for insulin tolerance tests (ITT) and Units of AUC for ITT of mice. n = 6. NC, DKD, MET, MV, and ML groups represent the normal control group treated with drug-free solution, type 2 diabetic kidney disease group treated with drug-free solution, DKD treated with metformin (MET) at a dose of 200 mg/kg/day, DKD treated with MET at a dose of 200 mg/kg/day and Valsartan (Val) at a dose of 30 mg/kg/day, and DKD treated with MET at a dose of 200 mg/kg/day and LW at a dose of 6.75 g/kg/day. Data are expressed as the mean ± SD. **P < 0.01 or ***P < 0.001 versus NC group; ^*#*^ *P* < 0.05, ^*##*^ *P* < 0.01, or ^*###*^ *P* < 0.001 versus DKD group; ^&^ *P* < 0.05, versus MET; ^$^ *P* < 0.05 or ^$$^ *P* < 0.01 versus MV.

Liuwei Dihuang concentrated pills (Batch No.: 2203158, 1.44g/8 pills, which is equivalent to 3 g of draw materials) were purchased from Lanzhou Foci Pharmaceutical Company (Lanzhou, China). The content was determined using the high-performance liquid chromatography (HPLC) method specified in the Pharmacopoeia of the People’s Republic of China (2020 Edition). The chromatogram is shown in Figure 1A, and the contents of morroniside, loganin, and paeonol are 0.90 mg, 0.69 mg, and 0.40 mg per pill, respectively, which complies with the requirements of the Pharmacopoeia of the People’s Republic of China (2020 Edition, Volume Ⅰ). Metformin hydrochloride (MET) (C16111825) and valsartan (Val) (C15474335) were purchased from Macklin Biochemical Technology Co., Ltd. (Shanghai, China). STZ (≥98%, HPLC grade) (WXBD1402V) was obtained from Sigma-Aldrich (St. Louis, MO, USA). Trizol Total RNA Extraction Reagent (99940205), Takara PrimeScript™ RT Master Mix (AO11067A), and Takara TB Green™ Premix Ex Taq™ II (AO11822A) were purchased from Takara Bio Inc. (Beijing, China). TG (A110-1-1), total cholesterol (TC) (A111-1-1), high-density lipoprotein cholesterol (HDL-C) (A112-1-1), low-density lipoprotein cholesterol (LDL-C) (A113-1-1), blood urea nitrogen (BUN) (C013-1-1), and creatinine (Cre) (C011-2-1) assay kits were obtained from Nanjing Jiancheng Bioengineering Institute (Nanjing, China). The ELISA kits (202,402) were purchased from Lampai Biotechnology Co., Ltd. (Shanghai, China). Mouse-specific primers (upstream and downstream) were bought from Sangon Biotech Co., Ltd. (Shanghai, China). Blood glucose test strips (1309100) were obtained from Jiangsu Yuyue Kailite Biotechnology Co., Ltd. (Jiangsu, China). RIPA buffer (P0013B), BCA Protein Assay Kit (60723231026), 5× sodium dodecyl sulfate (SDS)-polyacrylamide gel electrophoresis (PAGE) Loading Buffer (101623240220), and Western blot primary/secondary antibody diluents (P0023A and P0023D) were obtained from Beyotime Biotechnology (Shanghai, China). Protease/Phosphatase Inhibitor Cocktail (ST506 and 20034924) and SMAD7 Antibody (25,840-1-ap) were bought from Wuhan Sanying Biotechnology Co., Ltd. (Wuhan, China). The Fast PAGE Gel Preparation Kit (7E2380K4) was purchased from Vazyme Biotech Co., Ltd. (Nanjing, China). Nitrocellulose membranes (72147213) were provided by GE Healthcare (Chicago, IL, USA). Antibodies against GAPDH (ab181602), TGF-β (ab215715), and SMAD3 (ab40854) were purchased from Abcam Trading Co., Ltd. (Shanghai, China). Phospho-SMAD3 (p-SMAD3) antibody (C25A9) was purchased from Cell Signaling Technology (CST, USA). Goat anti-Rabbit/Mouse IgG (D40625-05), SDS-PAGE Electrophoresis Buffer Powder (VP6013P), and Tween-20/TBS Solution (20241010) were purchased from Xuzhou Microman Biotechnology Co., Ltd. (Jiangsu, China). The PageRuler™ Prestained Protein Marker (2969018) was purchased from Thermo Fisher Scientific (Waltham, Massachusetts, USA).

### 2.2 Animal experiment and sample collection

Forty 4-week-old male Sprague-Dawley (SD) rats weighing 200 ± 20 g were obtained from Beijing Sibefu Company (Beijing, China). The rats were housed in an SPF-grade animal facility with free access to water, and water bottles and bedding were changed daily. The room temperature was maintained at 22 °C ± 2 °C, with relative humidity of 55% ± 10%, under a 12 h light/12 h dark cycle. After a 1-week acclimatization period, the rats were randomly assigned to two groups based on body weight: normal control group (NC) and model group. The NC group received standard chow (10% fat, 20% protein, and 70% carbohydrate), while the model group received a high-fat diet (HFD; 45% fat, 20% protein, and 35% carbohydrate), which was obtained from Jiangsu GemPharmatech Co., Ltd. (Nanjing, China). At week 5, rats in the model group received a single intraperitoneal injection of STZ (35 mg/kg) dissolved in citrate buffer (0.1 mol/L, pH 4.5). NC rats received an equivalent volume of citrate buffer. After stable hyperglycemia was confirmed over 2 weeks, rats exhibiting fasting blood glucose (FBG) levels >11.1 mmol/L on two consecutive measurements were designated as T2DM models. The diabetic rats were further randomly divided into four groups based on FBG levels: DKD control group (DKD), DKD + metformin hydrochloride (MET, 200 mg/kg/day) intervention group, DKD + valsartan (30 mg/kg/day) + metformin hydrochloride (200 mg/kg/day) intervention group (MV), and DKD + LW (6.75 g/kg) + metformin hydrochloride (200 mg/kg/day) intervention group (ML). From week 7 onwards, the drug intervention groups received their respective treatment for 10 consecutive weeks. The NC and DKD control groups were administered equivalent volumes of ultrapure water. Body weight, food intake, and FBG levels were recorded weekly throughout the study period. Insulin tolerance tests (ITT) and oral glucose tolerance tests (OGTT) were conducted at week 16 and week 17, respectively.

At the experimental endpoint, fecal and 24-h urine samples were collected from all the groups. The rats were subsequently euthanized by exsanguination of the abdominal aorta to collect whole blood samples. Then, the kidneys were rapidly excised, rinsed thoroughly with ice-cold sterile saline, gently blotted dry with a filter paper, and weighed. Kidney tissues were dissected to the appropriate sizes on an ice platform. Portions of the tissues were immediately immersed in 4% paraformaldehyde fixative solution for subsequent Masson staining. The remaining tissue samples were flash-frozen in liquid nitrogen and then transferred to a −80 °C freezer for long-term storage, pending further molecular biology analyses.

A schematic of the experimental timeline is shown in Figure 1B. All the animal experimental protocols were approved by the Animal Ethics Committee of Xuzhou Medical University (Approval no. 202301T010).

### 2.3 Physiological and biochemical parameters assay

TC, TG, LDL-C, HDL-C, superoxide dismutase (SOD), malondialdehyde (MDA), catalase (CAT), glutathione peroxidase (GSH-Px), blood creatinine, and BUN in blood were measured using their corresponding commercial assay kits, following the manufacturer’s instructions. Cystatin C (Cys-C) in plasma and urinary creatinine (Ucr) and urinary microalbumin (MAU) levels in 24-h urine samples were quantified using corresponding ELISA kits. The urinary albumin/creatinine ratio (UACR) and urinary albumin excretion rate (UAER) were calculated using the following equations:

$$\text{UACR}=\text{MAU }\left(\mu g/\text{mL}\right)\times 1000/\text{Ucr }\left(\mu \text{mol}/L\right)$$

$$\text{UAER}=\text{MAU }\left(\mu g/\text{mL}\right)\times 24-h\text{ urine volume }\left(\text{mL}\right)$$

### 2.4 RT-PCR

Total RNA was isolated from renal cortical tissues using TRIzol Total RNA Extraction Reagent following the manufacturer’s protocol. RNA purity and concentration were determined spectrophotometrically (NanoDrop 1000; Thermo Fisher Scientific). First-strand cDNA was synthesized from 1 μg total RNA using a reverse transcription kit (Takara PrimeScript™ RT Master Mix) and stored at −20 °C. Reactions were performed using TB Green Premix Ex Taq™, II, with cycling conditions: 95 °C for 3 min, followed by 40 cycles of denaturation for 5 s at 95 °C, annealing and extension for 30 s at 65 °C. Primer sequences (Supplementary Material Supplementary Table S1) were designed to generate 80–150 bp amplicons. All samples were run in triplicate. The relative mRNA expression was calculated using the comparative 2^−ΔΔCt^ method: ΔCt = Ct (target gene) − Ct (GAPDH) and ΔΔCt = ΔCt (treatment group) − ΔCt (control group).

### 2.5 Renal tissue morphometric analysis

Kidney tissues were fixed in *4% paraformaldehyde* and subjected to gradient ethanol dehydration (70% → 100%), xylene clearing, and paraffin embedding. Serial sections (4-μm thickness) were cut using a rotary microtome, mounted on poly-lysine-coated slides, and dried at 60 °C for 2 h. After deparaffinization with xylene (10 min × 2), gradient ethanol rehydration (100% → 70%), and washing with distilled deionized H_2_O (ddH_2_O), Masson staining was performed. Images were captured using bright-field microscopy (Olympus BX43/DP73, Olympus Corporation, Tokyo, Japan) at ×200 magnification, and 10 random fields per section were analyzed using the ImageJ software (v1.53) with threshold-based color segmentation.

The formula for calculating relative collagen area percentage is expressed as follows:

$$\text{Relative collagen area }\left(\%\right)=\left(\text{collagen fiber area}/\text{total tissue area}\right)\times 100.$$

### 2.6 Western blot

Tissue homogenization was conducted using radioimmuno-precipitation assay lysis buffer supplemented with protease inhibitors to extract the total protein. The protein concentration was quantified using a Protein Assay Kit. Protein samples were normalized to a uniform concentration of 2 μg/μL by diluting with ddH_2_O, followed by addition of 5× SDS loading buffer and thorough vortexing. Denaturation was achieved by boiling the samples at 100 °C for 10 min. Protein separation was performed by SDS-PAGE. Subsequently, the proteins were transferred onto nitrocellulose membranes under wet transfer conditions. The membrane was blocked with rapid blocking buffer for 20 min at room temperature, then incubated with primary antibodies (TGF-β, SMAD7, p-SMAD3, SMAD3, and GAPDH as an internal reference) at 4 °C overnight with gentle agitation. The following day, the membrane was washed thrice with Tris-buffered saline containing 0.1% Tween-20 (TBST). The secondary antibody was incubated at room temperature for 1 h in the dark, and the membrane was washed thoroughly with TBST. Fluorescent signals were detected and scanned using an Odyssey dual-color infrared laser imaging system (LI-COR Corporate, Lincoln, Nebraska, USA). Band intensity was quantified by measuring gray values using the ImageJ software (v1.53) for densitometric analysis.

### 2.7 Fecal metabolomics

A pre-chilled 2-mL microcentrifuge tubes was loaded with 25 mg of feces, and homogenized beads were added to 500 μL extraction solvent (methanol-acetonitrile-water, 2:2:1, v/v) containing isotopically labeled internal standards. After vortex mixing at 2,500 rpm for 30 s, the mixture was homogenized at 35 Hz for 4 min (JXFSTPRP-24, Shanghai Jingxin Industrial Development Co., Ltd., Shanghai, China). Then, ultrasound-assisted extraction of the homogenate was performed in an ice-water bath (5 min × 3 cycles). Subsequently, the sample was incubated at −40 °C for 1 h and then centrifuged at 138,00 *g* at 4 °C for 15 min. The supernatant was collected in vials for ultra-high-performance liquid chromatography coupled with tandem mass spectrometry (UPLC-MS/MS) analysis. Equal volumes of supernatant from all samples were pooled to prepare quality control (QC) samples, which were then subjected to UPLC-MS/MS analysis.

The UPLC-MS/MS analysis of sample was carried out using a Vanquish UPLC system interfaced with an Orbitrap Exploris 120 mass spectrometer (Thermo Fisher Scientific, Waltham, Massachusetts, USA). Chromatographic separation was performed on a Waters ACQUITY UPLC BEH Amide column (2.1 mm × 50 mm, 1.7 μm particle size). The mobile phase consisted of an aqueous phase containing 25 mmol/L ammonium acetate and 25 mmol/L ammonium hydroxide (pH adjusted to 9.75) (A), and acetonitrile (B). A gradient elution program was applied as follows: 0–0.25 min, 5% A; 0.25–3.5 min, 5%–35% A (linear gradient); 3.5–4.0 min, 35%–60% A (linear gradient); 4.0–4.5 min, 60% A (isocratic); 4.5–4.55 min, 60%–5% A (linear gradient); and 4.55–6.0 min, 5% A (column re-equilibration). The flow rate was maintained at 0.5 mL/min. The sample tray temperature was set at 4 °C and the injection volume was 2 μL. The electrospray ionization (ESI) source was operated with a sheath gas flow rate of 50 Arb, auxiliary gas flow rate of 15 Arb, and capillary temperature set to 320 °C. The mass spectrometric parameters included a full-scan resolution of 60,000 and MS/MS resolution of 15,000. Collision energies of 20, 30, and 40 eV were applied in stepped normalized collision energy (SNCE) mode. The spray voltages were configured to +3.8 kV and −3.4 kV for positive and negative ionization modes, respectively.

Raw data were first converted to the mzXML format using ProteoWizard software, followed by mass spectrometry data preprocessing using the XCMS package (version 3.0) for peak detection, noise reduction, and alignment. Metabolite annotation was performed via an R-based pipeline, using BiotreeDB (version 3.0) as the reference database. For multivariate statistical analysis, processed data were imported into SIMCA (version 18.0.1) for principal component analysis (PCA) and orthogonal partial least squares discriminant analysis (OPLS-DA). To validate the robustness of the OPLS-DA model, a 200-permutation test was applied to evaluate goodness-of-fit parameters (R^2^ and Q^2^). Differential metabolites were screened using the following criteria: variable importance in projection (VIP) scores >1, *P* < 0.05, derived from Student’s t-test, and fold change (FC) thresholds of >2 or <0.5. After removing exogenous metabolites cross-referenced against the HMDB, PubChem, and LIPIDMAPS databases, pathway enrichment analysis was performed on the identified differential metabolites to elucidate the perturbed metabolic networks.

### 2.8 Metagenomics

Fecal microbial genomic DNA was isolated and purified using a Magnetic Bead-based Stool Genomic DNA Extraction Kit (AU46111-96; Bioteke Corporation, Beijing, China), according to the manufacturer’s protocol. DNA concentration was measured using a Qubit 1X dsDNA HS Assay Kit (Q33230, Invitrogen, Thermo Fisher Scientific, MA, USA). 200 ng of DNA meeting the QC requirements was taken into a 0.6 mL low-adsorption centrifuge tube (MCT-060-L-C, Axygen Biotechnology Co., Ltd., Hangzhou, China) and the volume was adjusted to 52 μL with water. The parameters of the sonicator (BioruptorTMPico, Diagenode Corporation, Seraing, Belgium) were adjusted to fragment the DNA to meet the fragment length requirement (200–500 bp). The fragmented products were purified and recovered using magnetic beads and TruSeq Library Construction Kit v2 (Illumina Inc., CA, USA). An Agilent 2,100 Bioanalyzer with High-sensitivity DNA Reagents (5067–4627) (Agilent Technologies Inc., CA, USA) was used to check whether the size of the fragmented products met the requirements. Samples that did not meet fragment range requirements were resampled and fragmented. The genomic library from the fragmented products was constructed using the TruSeq Nano DNA LT Library Preparation Kit (FC-121–4001, Illumina Inc., CA, USA) through end-repair, adapter ligation, index PCR amplification, and purification and quantified using Qubit 1X dsDNA HS Assay Kits. Finally, paired-end (PE) sequencing was performed on an Illumina Novaseq 6,000 using Illumina NovaSeq 6000 XP 4-Lane Kit v1.5 (300 cycles) (20043131) and sequencing mode of PE150.

The QC of raw sequencing was performed using the fastp software (v 0.23.4) with the parameters–l 100–g–W 6–5 -q 20–30. Bowtie 2 (v 2.2.0) was used to align the sequencing data with the host genome, and the sequences aligned to the host genome were filtered to ensure that the subsequent assembly and analysis results were microbial sequences. After obtaining valid sequences, MEGAHIT software (https://github.com/voutcn/megahit, v 1.2.9) was used to assemble the valid data of each sample to obtain contigs, and contigs with a length greater than 500 bp were retained. The coding DNA sequence (CDS) was predicted using MetaProdiga software (v. 2.6.3), and sequences with a CDS length less than 100 nt were filtered out. Subsequently, MMseqs2 software (v 15-6f452) was used for redundancy removal to obtain non-redundant unigenes. Clustering was performed with an identity of 95% and coverage of 90%, and the longest sequence was selected as the representative sequence to construct the unigene set. Bowtie2 was used to align the valid sequences of each sample to each unigene sequence and the number of reads aligned to each unigene in each sample was calculated. Unigenes with ≤2 aligned reads in all samples were filtered to obtain the final unigene set for subsequent analysis and to calculate the abundance of each unigene.

The unigenes were aligned with the NR_meta database using the DIAMOND software (v 0.9.14) to obtain species annotation information at different taxonomic levels. These sequences were also aligned with functional databases such as Kyoto Encyclopedia of Genes and Genomes (KEGG) (http://www.genome.jp/kegg/, v 87.1) and Evolutionary Genealogy of Genes: Non-supervised Orthologous Groups (eggNOG) (http://eggnogdb.embl.de/download/, v 5.0) to obtain annotation information for each functional database. Finally, based on the abundance statistics of unigenes, the abundance information of each species and functional classification level were obtained. A differential statistical analysis was conducted among the comparison groups based on species and function. Fisher’s exact test was used for the differential comparisons of samples without biological replicates. The Wilcoxon rank-sum test was used for differential comparisons between the two groups of samples with biological replicates. The Kruskal–Wallis test was used for comparisons among multiple groups of samples with biological replicates. The differential thresholds were set at *P* < 0.05 and log2(FC) > 1. Functional enrichment analysis was performed using ReporterScore (https://github.com/Asa12138/ReporterScore).

Alpha diversity indices, including Chao1, Observed species, Good coverage, Shannon index, and Simpson index, were calculated at the species level using QIIME1 (http://qiime.org/). The Wilcoxon rank-sum test was used for comparisons between two groups and the Kruskal–Wallis test was used for comparisons among multiple groups. Beta diversity was visualized by calculating Bray-Curtis distances and employing PCA, principal coordinate analysis (PCoA), and nonmetric multidimensional scaling (NMDS) with the R language (v 3.6.0).

Linear discriminant analysis (LDA) effect size (LEfSe) was used to analyze inter-group differences at various taxonomic levels, with a threshold of LDA >3.0 and *P* < 0.05, and the results were visualized using cladograms and bar charts. Differential analysis of species between the two groups at the species level was performed using R package metagenomeSeq (v 1.38.0).

### 2.9 Statistical analysis

Data are expressed as mean ± standard deviation (SD) and were analyzed using SPSS 26.0 (IBM Corporation, NY, USA). Inter-group comparisons were performed using the Student’s t-test for two-group analyses and one-way analysis of variance (ANOVA) for multi-group comparisons. The statistical significance threshold was set at α = 0.05, and two-tailed *P* < 0.05, were considered indicative of statistically significant differences.

## 3 Results

### 3.1 Effects of LW on FBG, body weight, 24 h urine, and renal index in DKD rats

As shown in Figure 1C, DKD rats exhibited significantly increased body weight compared to the NC group after 4 weeks of high-fat diet feeding (*P* < 0.01). Following STZ induction for model establishment, DKD rats demonstrated increased water intake, food consumption, and urine output, accompanied by reduced body weight, yellowish-moist fur, and elevated excretion of bedding materials. After 10 weeks of drug intervention, body weight gain in the DKD group was significantly lower than that in the NC group (*P* < 0.001), whereas the ML treatment group showed significantly enhanced body weight gain compared to the DKD group (*P* < 0.001) and demonstrated superior efficacy compared to the MET and MV groups (*P* < 0.05) (Figure 1D). As shown in Figure 1E, the renal index of DKD rats was significantly elevated compared to that of the NC group (*P* < 0.001). All treatment groups exhibited significant reductions in renal index relative to the DKD group (*P* < 0.01 or 0.001). Notably, ML demonstrated superior efficacy in improving the renal index compared to MET (*P* < 0.05).

As illustrated in Figure 1F, throughout the experimental period, FBG levels in the DKD group were significantly higher than those in the NC group (*P* < 0.001). The ML group exhibited a significant reduction in FBG at the third week (*P* < 0.01), with values significantly lower than those in the MET group at the sixth week (*P* < 0.05) and significantly lower than those in the MV group at the fourth, sixth, and eighth weeks (*P* < 0.05). No significant differences in FBG levels were observed between the MET and MV groups throughout the study period. From the sixth week onwards, all drug-treated groups showed significantly lower FBG levels than those in the DKD group (*P* < 0.01 or 0.001).

As shown in Figures 1G–I, at the end of the experiment (17th week), the DKD group displayed significantly elevated FBG levels, fasting plasma insulin levels, and the homeostatic model assessment of insulin resistance (HOMA-IR) index compared to the NC group (*P* < 0.001). All drug intervention groups exhibited significantly reduced FBG and HOMA-IR indices compared to the DKD group (*P* < 0.001). The MET and MV groups demonstrated a significant decrease in fasting plasma insulin levels (*P* < 0.01), whereas ML had no effect on fasting plasma insulin levels in DKD rats. Notably, MV showed superior efficacy in ameliorating fasting plasma insulin levels compared to ML (*P* < 0.05). These results suggest that MET monotherapy or its combination with LW and Val can improve blood glucose levels and IR in rats with DKD. Consistent with these findings, OGTT and ITT results (Figures 1J,K) revealed that MET, alone or in combination with LW and Val, effectively enhanced glucose tolerance and insulin sensitivity in DKD rats.

### 3.2 Effects of LW on lipid profiles and oxidative stress in DKD rats

**FIGURE 2:**
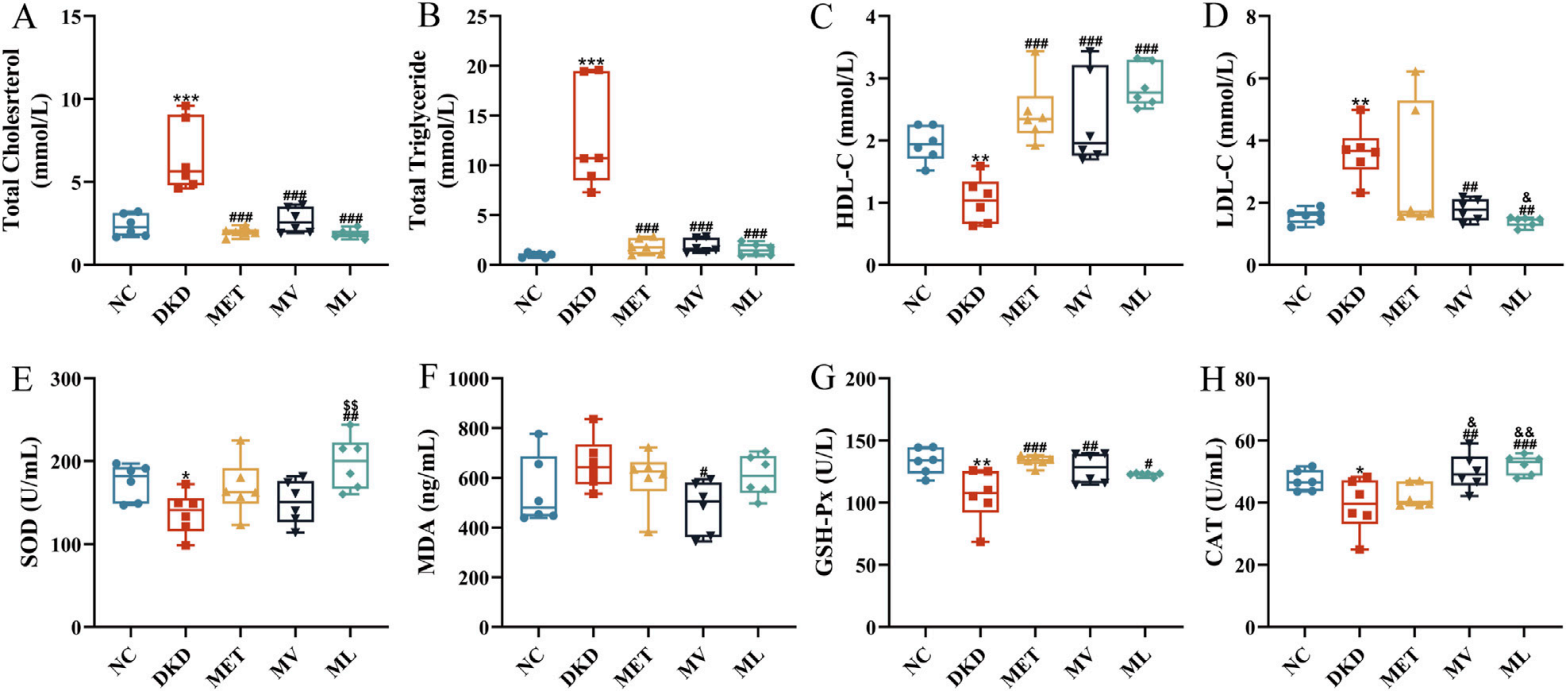
**(A)** Plasma total cholesterol (TC), **(B)** Plasma triglyceride (TG), **(C)** Plasma high-density lipoprotein cholesterol (HDL-C), **(D)** Plasma low-density lipoprotein cholesterol (LDL-C), **(E)** Plasma superoxide dismutase (SOD), **(F)** Plasma malondialdehyde (MDA), **(G)** Plasma glutathione peroxidase (GSH-Px), and **(H)** Plasma catalase (CAT). n = 6. NC, DKD, MET, MV, and ML groups represent the normal control group treated with drug-free solution, type 2 diabetic kidney disease group treated with drug-free solution, DKD treated with metformin (MET) at a dose of 200 mg/kg/day, DKD treated with MET at a dose of 200 mg/kg/day and Valsartan (Val) at a dose of 30 mg/kg/day, and DKD treated with MET at a dose of 200 mg/kg/day and LW at a dose of 6.75 g/kg/day. Data are expressed as the mean ± SD. *P < 0.05, **P < 0.01, or ***P < 0.001 versus NC group; ^*#*^ *P* < 0.05, ^*##*^ *P* < 0.01, or ^*###*^ *P* < 0.001 versus DKD group; ^&^ *P* < 0.05 or ^&&^ *P* < 0.01 versus MET; ^$$^ *P* < 0.01 versus MV.

As shown in Figures 2A–D, compared with the NC group, the DKD group exhibited significantly elevated plasma levels of TC, TG, and LDL-C (*P* < 0.001 or 0.01), along with a marked reduction in HDL-C (*P* < 0.01). In contrast, the MET group demonstrated a significant decrease in TC and TG levels (*P* < 0.001) and a notable increase in HDL-C levels (*P* < 0.001) relative to the DKD group. Both the MV and ML groups displayed significantly reduced plasma TC, TG, and LDL-C levels (*P* < 0.001 or 0.01) and increased HDL-C levels (*P* < 0.001) compared to the DKD group. These findings indicate that MET monotherapy or its combination with Val and LW ameliorates dyslipidemia in rats with DKD, with combination therapies yielding superior efficacy compared to MET alone.

As shown in Figures 2E–H, relative to the NC group, the DKD group showed significantly decreased plasma levels of SOD, GSH-Px, and CAT (*P* < 0.05 or 0.01), whereas MDA levels were elevated without statistical significance. MET monotherapy significantly increased the plasma GSH-Px levels (*P* < 0.001), whereas the MV group exhibited enhanced plasma GSH-Px and CAT levels (*P* < 0.01) and reduced MDA levels (*P* < 0.05). Notably, MV surpassed MET in improving the plasma CAT activity (*P* < 0.05). The ML group showed pronounced upregulation of plasma SOD, GSH-Px, and CAT levels (*P* < 0.001, 0.01, or 0.05), with its SOD-enhancing effect being significantly greater than that of MV (*P* < 0.01) and its CAT-enhancing effect surpassing that of MET (*P* < 0.05). These results confirmed that combining MET with LW or Val synergistically alleviated oxidative stress in rats with DKD.

### 3.3 Effects of LW on renal function, inflammation, and morphology in DKD rats

**FIGURE 3:**
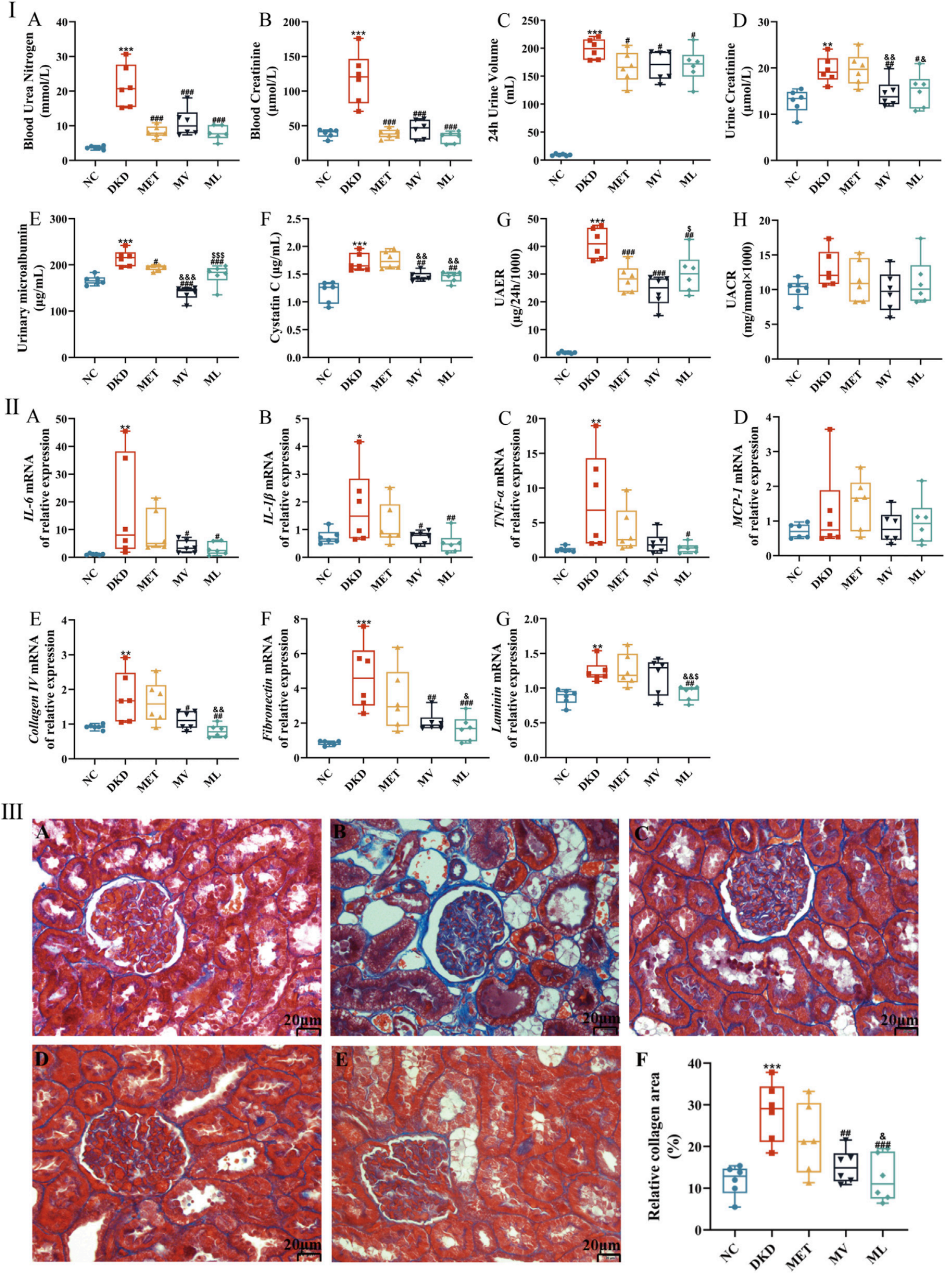
**(Ⅰ)**: **(A)** Blood urea nitrogen, **(B)** Blood creatinine, **(C)** 24 h urine volume, **(D)** Urine creatinine, **(E)** Urinary microalbumin, **(F)** Cystatin C, **(G)** Urinary albumin excretion (UAER), and **(H)** Urinary microalbumin and urine creatinine ratio (UACR). **(Ⅱ)**: **(A)** Renal *interleukin (IL)-6* mRNA expression, **(B)** Renal *IL-1*β mRNA expression, **(C)** Renal *tumor necrosis factor α (TNF-*α*)* mRNA expression, **(D)** Renal *monocyte chemoattractant protein-1 (MCP-1)* mRNA expression, **(E)** Renal *collagen Ⅳ* mRNA expression, **(F)** Renal fibronectin mRNA expression, and **(G)** Renal laminin expression. **(Ⅲ)**: histopathological images (magnification ×200) of kidney in NC **(A)**, DKD **(B)**, MET **(C)**, MV **(D)**, ML **(E)**, and the relative collagen area **(F)**. n = 6. NC, DKD, MET, MV, and ML groups represent the normal control group treated with drug-free solution, type 2 diabetic kidney disease group treated with drug-free solution, DKD treated with metformin (MET) at a dose of 200 mg/kg/day, DKD treated with MET at a dose of 200 mg/kg/day and Valsartan (Val) at a dose of 30 mg/kg/day, and DKD treated with MET at a dose of 200 mg/kg/day and LW at a dose of 6.75 g/kg/day. Data are expressed as the mean ± SD. *P < 0.05, **P < 0.01, or ***P < 0.001 versus NC group; ^*#*^ *P* < 0.05, ^*##*^ *P* < 0.01, or ^*###*^ *P* < 0.001 versus DKD group; ^&^ *P* < 0.05, ^&&^ *P* < 0.01, or ^&&&^ *P* < 0.001 versus MET; ^$^ *P* < 0.05 or ^$$$^ *P* < 0.001 versus MV.

As illustrated in Figure 3IA,B, plasma levels of BUN and Cre were significantly elevated in DKD rats compared to those in the NC group (*P* < 0.01 or 0.001). BUN and Cre levels were significantly reduced in all the treatment groups (*P* < 0.001). Figure 3IC reveals that 24-h urine volume in the DKD group was markedly increased compared to the NC group rats (*P* < 0.001). After 10 weeks of drug intervention, all treatment groups exhibited significantly decreased 24-h urine volume compared to the DKD group (*P* < 0.05). Correspondingly, Figure 3ID-H demonstrate that Ucr, MAU, Cys-C, and UAER levels were significantly higher in DKD rats than in NC rats (*P* < 0.05 or 0.01). MET monotherapy only reduced MAU and UAER levels (*P* < 0.05 or 0.01), whereas all four urinary biomarkers were significantly lower in both the MV and ML groups (*P* < 0.05 or 0.01). Notably, MV outperformed MET in modulating Ucr, MAU, and Cys-C (*P* < 0.01 or 0.001), and surpassed ML in regulating MAU and Cys-C. Conversely, ML exhibited superior effects on MET in reducing Ucr and Cys-C levels (*P* < 0.05 or 0.01).

As shown in Figure 3IIA-D, mRNA levels of renal pro-inflammatory cytokines (*interleukin* (*IL*)*-6*, *IL-1*β, *tumor necrosis factor-α* (*TNF-*α)) were significantly upregulated in the DKD group compared to the NC group (*P* < 0.05 or 0.01). MV treatment notably reduced renal *IL-6* and *IL-1*β mRNA levels (*P* < 0.05), whereas ML treatment suppressed *IL-6*, *IL-1*β, and *TNF-*α mRNA expression (*P* < 0.05 or 0.01). Figure 3IIE-G indicates that renal mRNA levels of extracellular matrix (ECM) components, such as *collagen Ⅳ* (*Col Ⅳ*), *fibronectin* (*FN*), and *laminin* (*Lam*), were significantly elevated in DKD rats (*P* < 0.01 or 0.001). MV reduced *Col Ⅳ* and *FN* mRNA expression (*P* < 0.05 or 0.01), whereas ML downregulated *Col Ⅳ*, *FN*, and *Lam* mRNA levels (*P* < 0.01 or 0.001), surpassing both MV and MET (*P* < 0.05 or 0.01). These results suggest that LW or Val combined with MET can effectively attenuate renal inflammation and ECM accumulation in DKD rats.

Morphological findings further corroborated these results (Figure 3IIIA-F). NC rats exhibited normal glomerular and tubular structures, with negligible collagen deposition. In contrast, rats with DKD displayed mild glomerular atrophy, thickened basement membranes, tubular dilation, pronounced collagen staining, and severe fibrosis. The MET group showed reduced tubular dilation and persistent collagen deposition. Both MV and ML groups exhibited preserved glomerular architecture, alleviated tubular dilation, and significantly reduced collagen deposition (*P* < 0.01 or 0.001). Moreover, ML demonstrated a greater reduction in collagen deposition than MET (*P* < 0.05).

### 3.4 Effects of LW on the TGF-β/SMAD signaling pathway in the kidneys of DKD rats

**FIGURE 4:**
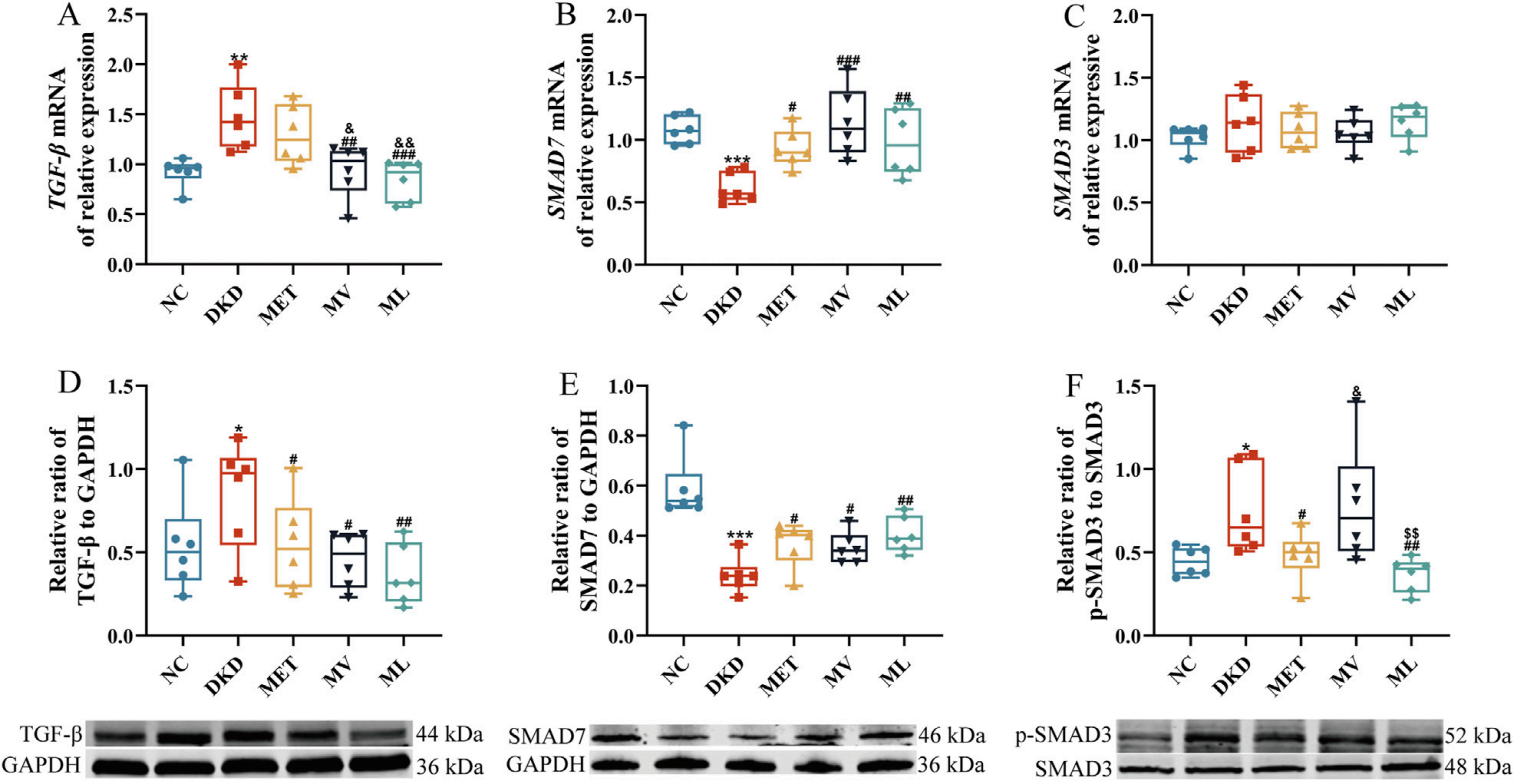
**(A)** Renal *transforming growth factor-β (TGF-β)* mRNA expression, **(B)** Renal *SMAD7* expression, **(C)** Renal *SMAD3* mRNA expression, **(D)** Renal TGF-β protein expression, **(E)** Renal SMAD7 protein expression, and **(F)** Renal phospho-SMAD3 (p-SMAD3) protein expression. n = 6. NC, DKD, MET, MV, and ML groups represent the normal control group treated with drug-free solution, type 2 diabetic kidney disease group treated with drug-free solution, DKD treated with metformin (MET) at a dose of 200 mg/kg/day, DKD treated with MET at a dose of 200 mg/kg/day and Valsartan (Val) at a dose of 30 mg/kg/day, and DKD treated with MET at a dose of 200 mg/kg/day and LW at a dose of 6.75 g/kg/day. Data are expressed as the mean ± SD. *P < 0.05, **P < 0.01, or ***P < 0.001 versus NC group; ^*#*^ *P* < 0.05, ^*##*^ *P* < 0.01, or ^*###*^ *P* < 0.001 versus DKD group; ^&^ *P* < 0.05 or ^&&^ *P* < 0.01 versus MET; ^$$^ *P* < 0.01 versus MV.

As shown in Figures 4A–C, compared to the NC group, the relative mRNA expression of *TGF-β* was significantly upregulated (*P* < 0.01), whereas *SMAD7* mRNA levels were markedly downregulated (*P* < 0.001) in the renal tissues of DKD rats. *SMAD7* mRNA levels were significantly elevated in the MET group compared to the DKD group. Both the MV and ML groups exhibited significant reductions in *TGF-β* mRNA expression (*P* < 0.01 or 0.001) and increased in *SMAD7* mRNA levels (*P* < 0.001) compared to the DKD group. Notably, *TGF-β* mRNA expression in the ML and MV groups was significantly lower than that in the MET group (*P* < 0.05 or 0.01). No statistically significant differences in *SMAD3* mRNA levels were observed between groups.

Western blot results (Figures 4D–F) demonstrated that the protein expression of TGF-β and p-SMAD3 was significantly increased (*P* < 0.05), whereas SMAD7 protein levels were markedly decreased (*P* < 0.001) in DKD rats compared to the NC group. All treatment groups effectively reversed the aberrant expression of TGF-β and SMAD7 proteins (*P* < 0.05 or 0.01) compared to the DKD group. Furthermore, the MET and ML groups significantly reduced *p*-SMAD3 protein expression (*P* < 0.05 or 0.01), with ML outperforming MV (*P* < 0.05 or 0.01).

Collectively, these findings indicate that LW combined with MET synergistically ameliorates TGF-β/SMAD signaling pathway dysregulation in the kidneys of DKD rats with superior efficacy compared to the combination of Val and MET.

### 3.5 Effects of LW on fecal metabolites in DKD rats

As shown in Supplementary Material Supplementary Table S2 and Supplementary Figures S1-S9, the UPLC-MS/MS method employed for untargeted metabolomics in this study demonstrated stable and reliable performance. A PCA score plot (Supplementary Figure S10) revealed a clear separation between the NC and DKD groups, indicating significant metabolic alterations in DKD rats. Partial separation was observed between the DKD and the MET or MV groups, whereas the ML group exhibited complete separation from the DKD group. These results suggest that treatment interventions modulate fecal metabolite profiles in DKD rats, with the combination of LW and MET exerting a more pronounced effect on metabolic reprogramming than MET alone or the combination of Val and MET.

**FIGURE 5:**
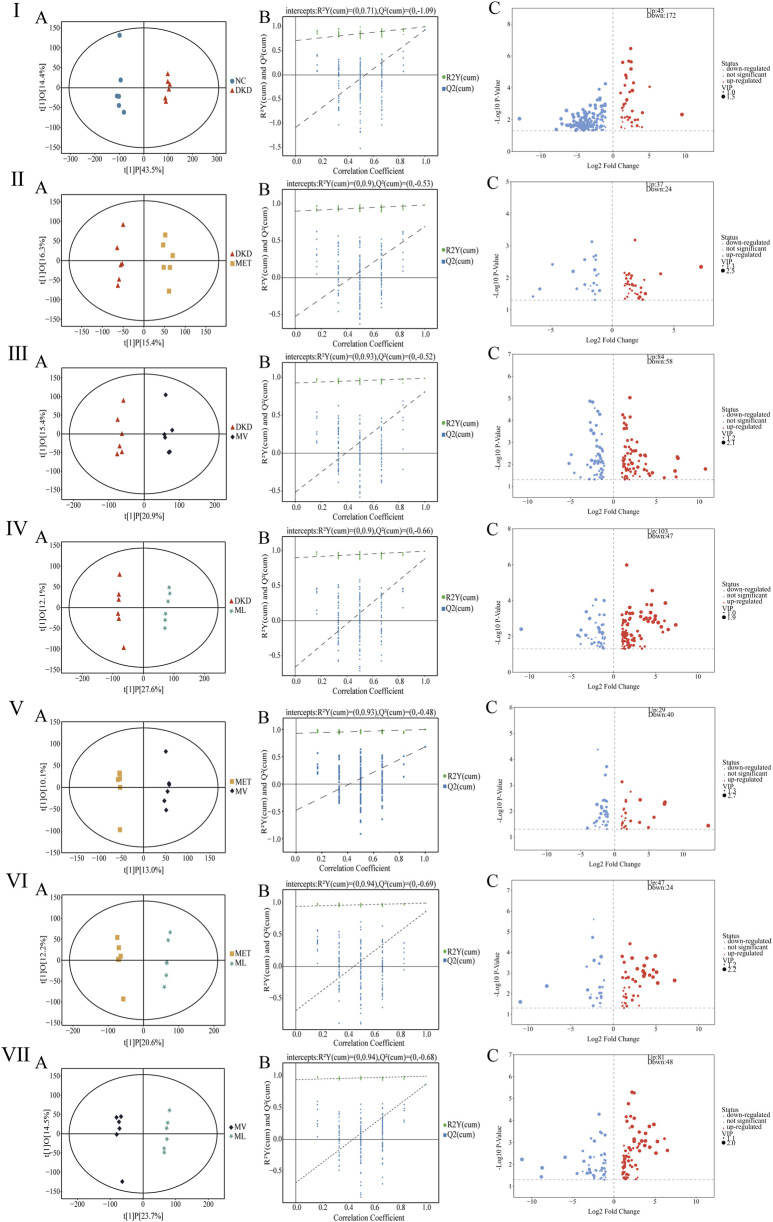
Fecal metabolites analysis in rats. **(Ⅰ)** **(A–C)**, **(Ⅱ)** **(A–C)**, **(Ⅲ)** **(A–C)**, **(Ⅳ)** **(A–C)**, **(Ⅴ)** **(A–C)**, **(Ⅵ)** **(A–C)**, and **(Ⅶ)** **(A–C)** represent orthogonal partial least squares discriminant analysis (OPLS-DA) plot, permutation test analysis of OPLS-DA model, and volcano plot between DKD group versus NC group, MET group versus DKD group, MV group versus DKD group, ML group versus DKD group, MV group versus MET group, ML group versus MET group, and ML group versus MV group, respectively. n = 6. NC, DKD, MET, MV, and ML groups represent the normal control group treated with drug-free solution, type 2 diabetic kidney disease group treated with drug-free solution, DKD treated with metformin (MET) at a dose of 200 mg/kg/day, DKD treated with MET at a dose of 200 mg/kg/day and Valsartan (Val) at a dose of 30 mg/kg/day, and DKD treated with MET at a dose of 200 mg/kg/day and LW at a dose of 6.75 g/kg/day.

The OPLS-DA results (Figures 5I–VIIA) demonstrated distinct separations among all comparative groups (NC vs. DKD, DKD vs. MET, DKD vs. MV, DKD vs. ML, MET vs. MV, MET vs. ML, and MV vs. ML), confirming substantial metabolic differences. A permutation test with 200 iterations (Figures 5I–VIIIB) validated the robustness of the OPLS-DA models, as the original R^2^ and Q^2^ values (both >0.5) for all models remained higher than those of the randomized permutations, ruling out overfitting.

After excluding exogenous metabolites and applying the criteria (VIP >1 and *P* < 0.05), differential metabolites were identified between the groups: NC vs. DKD, 217 metabolites (45 upregulated and 172 downregulated); DKD vs. MET, 61 metabolites (37 upregulated and 24 downregulated); DKD vs. MV, 142 metabolites (84 upregulated and 58 downregulated); DKD vs. ML, 150 metabolites (103 upregulated and 47 downregulated); MET vs. MV, 69 metabolites (29 upregulated and 40 downregulated); MET vs. ML, 71 metabolites (47 upregulated and 24 downregulated); MV vs. ML, 129 metabolites (81 upregulated and 48 downregulated) (Figures 5I–VIIIC). Further filtering (FC > 2 or <0.5) revealed distinct modulatory effects. As shown in Supplementary Material Supplementary Tables S3,S4, MET uniquely modulated tauro-gamma-muricholic acid, tauroursodeoxycholic acid, 16alpha,17beta-estriol-16-(beta-D-glucuronide) (three metabolites); MV uniquely modulated 2-methylbutyrylglycine, isobutyrylglycine, 3-dehydroquinic acid, cytosine, deoxyribose, 11-keto-beta-boswellic acid, 9,11-octadecadienoic acid, corchorusoside A, and others (10 metabolites); ML uniquely modulated 2-aminoisobutyric acid, euscaphic acid, ligustrazine, 3-amino-4-hydroxybenzoic acid, N-acetylneuraminic acid, N-acetyl-L-glutamate_5-semialdehyde, histidylproline diketopiperazine, and others (9 metabolites). In summary, MET alone minimally influenced the metabolic profile of DKD rats, whereas its combination with Val (MV) or LW (ML) induced significant metabolic reprogramming. Notably, MV and ML exhibited comparable efficacy in modulating fecal metabolites, highlighting the therapeutic potential of LW in synergizing with MET to restore metabolic homeostasis in DKD subjects.

**FIGURE 6:**
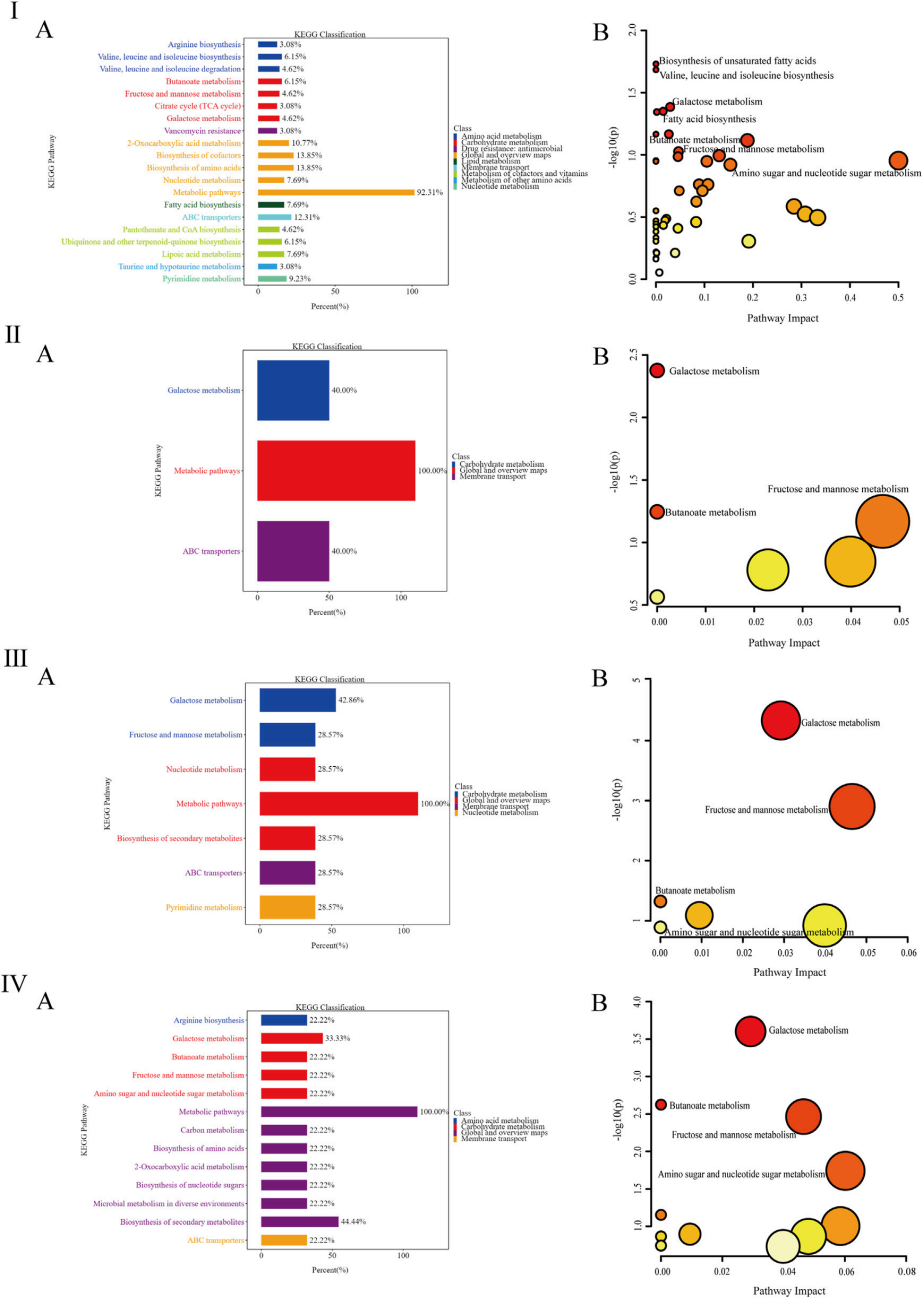
**(Ⅰ-Ⅳ)** represent the column diagram **(A)** and bubble diagram **(B)** of pathways in the Kyoto Encyclopedia of Genes and Genomes (KEGG) between the DKD group and the NC group, the MET group and the DKD group, the MV group and the DKD group, and the ML group and the DKD group, respectively. n = 6. NC, DKD, MET, MV, and ML groups represent the normal control group treated with drug-free solution, the type 2 diabetic kidney disease group treated with drug-free solution, DKD treated with metformin (MET) at a dose of 200 mg/kg/day, DKD treated with MET at a dose of 200 mg/kg/day, valsartan (Val) at a dose of 30 mg/kg/day, and DKD treated with MET at a dose of 200 mg/kg/day and LW at a dose of 6.75 g/kg/day.

The KEGG analysis results (Figure 6I-IVA indicated that the differentially metabolized pathways between the NC and DKD groups were primarily enriched in amino acid metabolism, biosynthesis, cofactors, cofactor biosynthesis, ABC transporters, and two-oxocarboxylic acid metabolism. The DKD and MET groups showed fewer differentially metabolized pathways, with enrichment only for galactose metabolism and ABC transporters. The differentially metabolized pathways between the DKD and MV groups were mainly enriched in galactose metabolism, fructose and mannose metabolism, nucleotide metabolism, secondary metabolite biosynthesis, ABC transporters, and pyrimidine metabolism. Compared to the MET and MV groups, the returned differentially metabolized pathways between the DKD and ML groups were enriched in a greater number of metabolic pathways. These results suggest that the differentially metabolized pathways returned by the combination of LW and MET can enrich metabolic pathways and regulate metabolism in DKD rats to the level of normal rats through multiple pathways.

To further analyze the impact of differentially metabolized pathways on their respective pathways, the differentially metabolized pathways were inputted into the MetaboAnalyst platform (http://www.metaboanalyst.ca/) with the species set to rats, and the analysis results were presented through a visual bubble chart. By integrating two dimensions of enrichment significance (*P*-value) and topological features (Impact value), as shown in Figure 6I-IVB, there are four significantly different metabolic pathways between the NC and DKD groups, which are the biosynthesis of unsaturated fatty acids, the biosynthesis of valine, leucine, and isoleucine, galactose metabolism, and fatty acid biosynthesis. The metabolic pathway that differed significantly between the DKD and MET groups was galactose metabolism. The significantly different metabolic pathways between the DKD and MV groups were galactose, fructose, mannose, and butyrate metabolisms. The significantly different metabolic pathways between the DKD and ML groups were galactose, butyrate, fructose and mannose, and amino and nucleotide sugar metabolism. The three treatment groups all had the galactose metabolism pathway, the MV and ML groups shared butyrate metabolism, fructose and mannose metabolism, and amino sugar and nucleotide sugar metabolism unique to the ML group. The above results suggest that butyrate metabolism and fructose and mannose metabolism may be key to the superior improvement effect of LW or Val combined with MET compared with the use of MET alone.

### 3.6 Impact of LW on the gut microbiota of DKD rats

The QC and assembly information for metagenomic sequencing of all samples is presented in Supplementary Tables S5, S6. The data in these tables indicate that the sequencing results of the samples in this study are reliable and can represent the microbial community composition of each sample for subsequent analyses.

**FIGURE 7:**
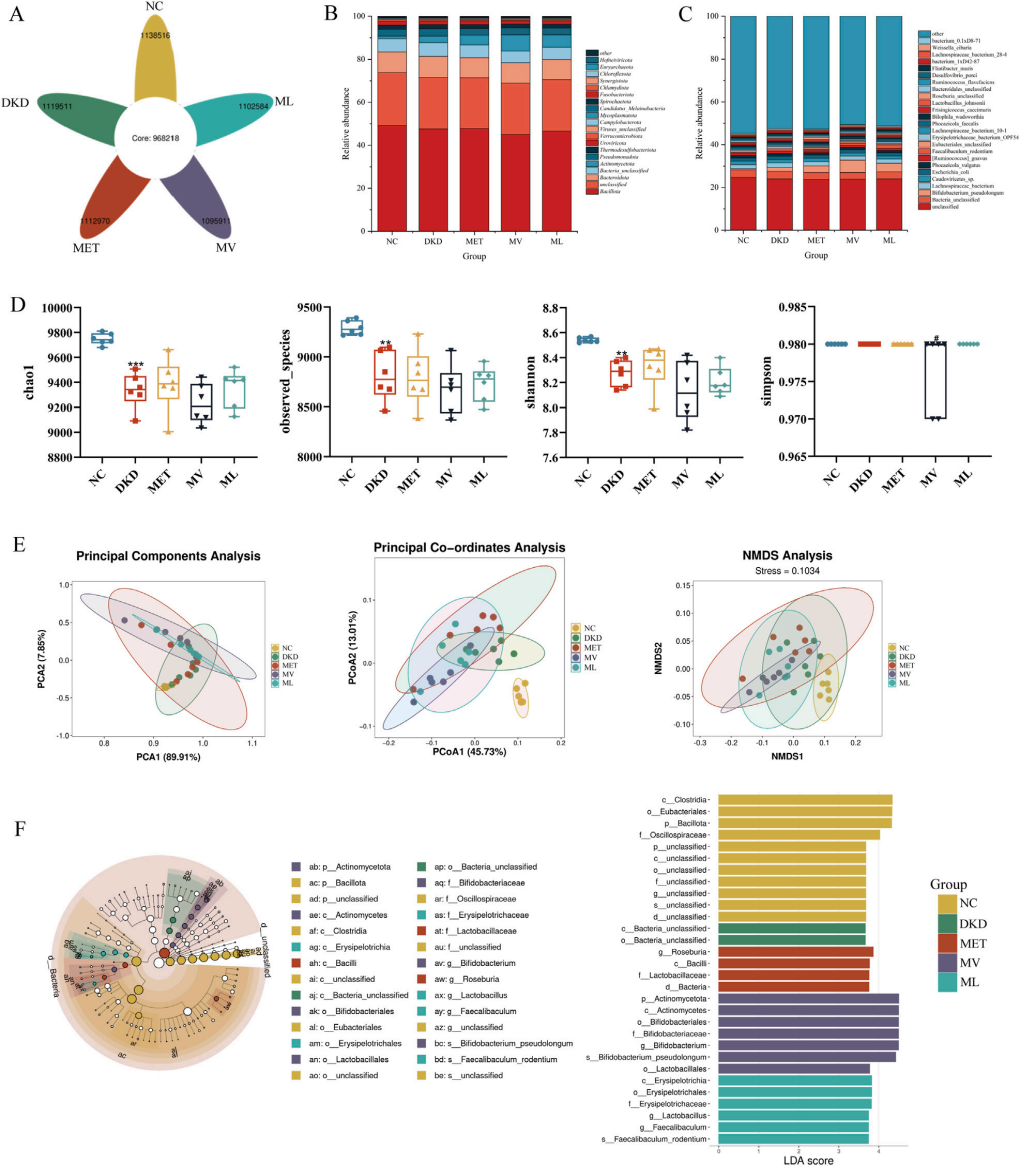
**(A)** Flower petal plot of Amplicon Sequence Variants (ASVs), **(B)** relative abundance of gut microbiota at the phylum level, **(C)** relative abundance of gut microbiota at the species level, **(D)** alpha-diversity analysis including chao1, observed_otus, shannon, and ssimpson of gut microbiota, **(E)** beta-diversity analysis including principal component analysis (PCA), principal coordinate analysis (PCoA), and nonmetric multidimensional scaling (NMDS) of gut microbiota, and **(F)** MetaStats analysis (Left), in which the diameter of each circle reflects the abundance of taxa in the community, and linear discriminant analysis effect size (LEfSe) sequence analysis (Right) across five experimental groups of rat fecal samples. n = 6. NC, DKD, MET, MV, and ML groups represent the normal control group treated with drug-free solution, type 2 diabetic kidney disease group treated with drug-free solution, DKD treated with metformin (MET) at a dose of 200 mg/kg/day, DKD treated with MET at a dose of 200 mg/kg/day and Valsartan (Val) at a dose of 30 mg/kg/day, and DKD treated with MET at a dose of 200 mg/kg/day and LW at a dose of 6.75 g/kg/day. Data are expressed as the mean ± SD. **P < 0.01, or ***P < 0.001 versus NC group; ^*#*^ *P* < 0.05 versus DKD group.

The amplicon sequence variants (ASVs) petal diagram of fecal samples from five groups of rats (Figure 7A) showed that there were a total of 5,569,492 ASVs across all fecal samples, with 968,218 shared ASVs accounting for 17.38% of the total ASVs. The numbers of ASVs unique to each group are as follows: NC group with 1,138,516 (20.44%), DKD group with 1,119,511 (20.10%), MET group with 1,112,970 (19.98%), MV group with 1,095,911 (19.68%), and ML group with 1,102,584 (19.80%).

A total of 160 phyla and 11,125 species of the gut microbiota were identified in this study. As shown in Figure 7B, at the phylum level, *Bacillota*, *Bacteroidota*, and *Bacteria_unclassified* were dominant in all five groups, while *Actinomycetota* was less abundant in the NC group but more abundant in the other groups, particularly in the MV group. At the species level (Figure 7C), *Bacteria_unclassified*, Lachnospiraceae*_bacterium*, *Caudoviricictes_sp*, *Escherichia_coli*, and *Phocaeicola_vulgatus* were dominant in all the five groups. *Bifidobacterium_pseudolongum* was the dominant species in the DKD, MET, MV, and ML groups, whereas *Faecalibaculum_rodentium* was relatively more abundant in the MV group. These results suggest that the gut microbiota of DKD rats is disrupted and that the MET, MV, and ML groups can exert regulatory effects by acting on different microbial communities.

Alpha diversity indices (Figure 7D) revealed that the Chao1, Observed_species, and Shannon indices in the DKD group were significantly lower than those in the NC group (*P* < 0.01 or 0.001). There were no significant differences in these indices between the DKD and the treatment groups. The Simpson index was significantly lower in the MV group than in the DKD group (*P* < 0.05). These results suggest that the gut microbiota of DKD rats exhibited a significant decrease in species richness and diversity compared to the NC group. Beta diversity analyses (PCA, PCoA, and NMDS) (Figure 7E) indicated that the NC and DKD groups were almost completely separated. Although the treatment groups were not fully separated from the DKD group, they exhibited varying degrees of divergence, and were closer to each other. This suggests that there is a significant difference in the microbial composition structure between the NC and DKD groups, and that the treatment groups may have altered the microbial community structure in the DKD group, potentially contributing to their therapeutic effects.

LEfSe analysis (Figure 7F) showed that *Clostridia* at the order level, *Eubacteriales* at the family level, *Bacillota* at the phylum level, and Oscillospiraceae at the genus level were relatively abundant in the NC group. In the DKD group, *Bacteria_unclassified* bacteria were highly abundant at both class and order levels. In the MET group, *Roseburia* at the genus level, *Bacilli* at the phylum level, Lactobacillaceae at the family level, and *Bacteria* at the domain level were relatively abundant. In the MV group, *Actinomycetota* at both the phylum and class levels; *Bifidobacteriales* at the order, family, and genus levels; *Bifidobacterium_pseudolongum* at the species level; and *Lactobacillales* at the order level were dominant. In the ML group, *Erysipelotrichia* at the class, order, and family levels; *Lactobacillus* at the genus level; *Faecalibaculum* at the genus level; and *Faecalibaculum_rodentium* at the species level were relatively abundant.

**FIGURE 8:**
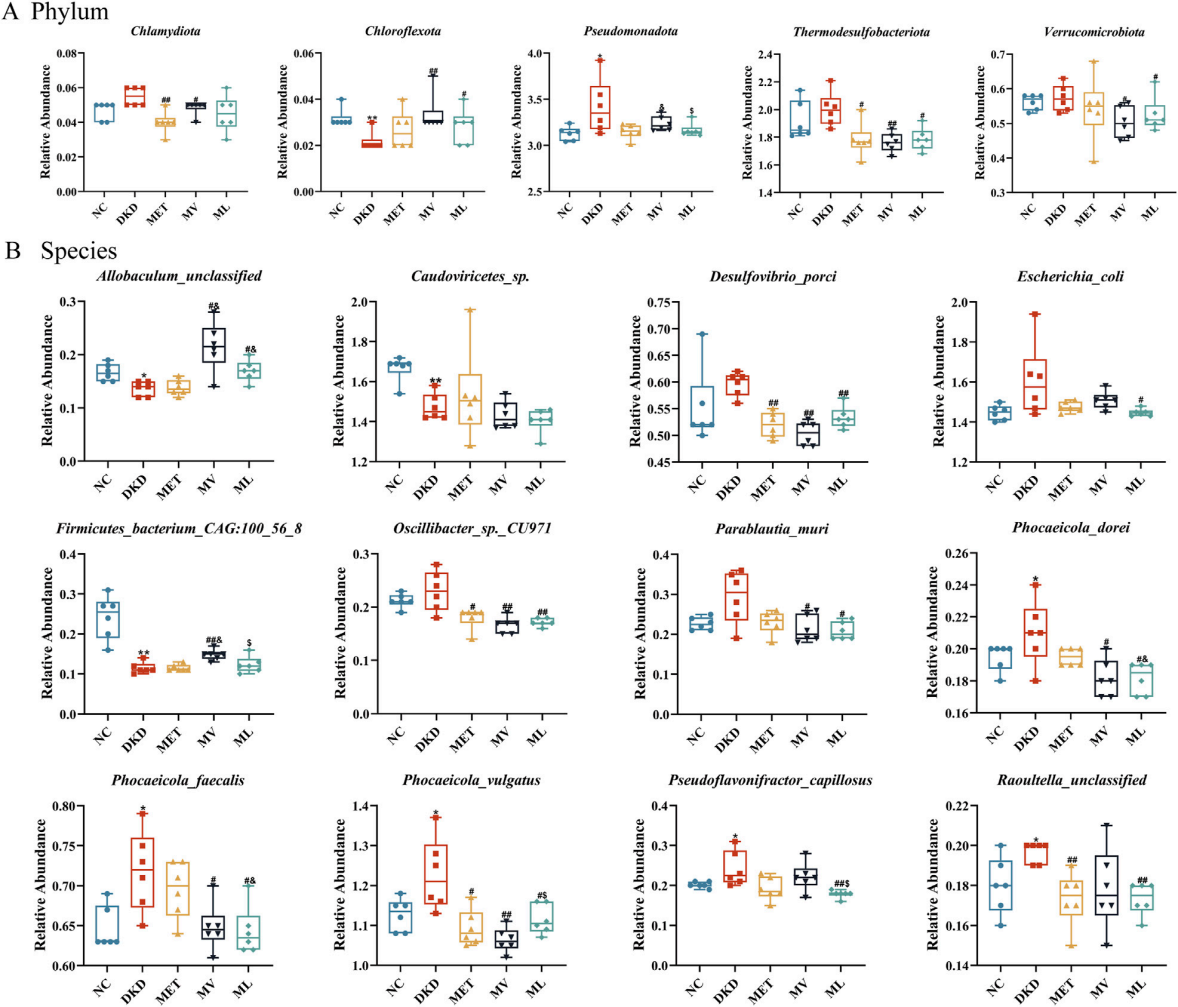
**(A)** The relative abundance of differential phylum and **(B)** the relative abundance of differential species across five experimental groups of rat fecal samples by unpaired t-test. n = 6. NC, DKD, MET, MV, and ML groups represent the normal control group treated with drug-free solution, type 2 diabetic kidney disease group treated with drug-free solution, DKD treated with metformin (MET) at a dose of 200 mg/kg/day, DKD treated with MET at a dose of 200 mg/kg/day and Valsartan (Val) at a dose of 30 mg/kg/day, and DKD treated with MET at a dose of 200 mg/kg/day and LW at a dose of 6.75 g/kg/day. Data are expressed as the mean ± SD. *P < 0.05 or **P < 0.01 versus NC group; ^*#*^ *P* < 0.05 or ^*##*^ *P* < 0.01 versus DKD group; ^&^ *P* < 0.05 versus MET; ^$^ *P* < 0.05 versus MV.

MetaStats analysis revealed significant alterations in the gut microbiome composition at the phylum level among the different treatment groups (Figure 8A). In the DKD group, the relative abundance of *Chloroflexota* was significantly reduced (*P* < 0.01), whereas the abundance of *Pseudomonadota* was markedly increased (*P* < 0.05) compared to the NC group. MET treatment significantly decreased the abundance of *Chlamydiota* and *Thermodesulfobacteriota* in the feces (*P* < 0.05 or 0.01). The MV group showed a significantly increased abundance of *Chloroflexota* (*P* < 0.01) and decreased abundance of *Chlamydiota*, *Thermodesulfobacteriota*, and *Verrucomicrobiota* (*P* < 0.05 or 0.01). ML treatment not only increased the abundance of *Thermodesulfobacteriota* (*P* < 0.05), but also modulated the abundance of *Chloroflexota* and *Verrucomicrobiota* (*P* < 0.05), which were not affected by MET alone. Furthermore, the reduction in *Pseudomonadota* abundance by ML was significantly more pronounced than that by MV (*P* < 0.05). At the species level (Figure 8B), compared to the NC group, the DKD group exhibited a significant decrease in the abundance of *Allobaculum_unclassified*, *Caudoviricetes_*sp., and *Firmicutes_bacterium_CAG:100_56_8* (*P* < 0.05 or 0.01) and a marked increase in the abundance of *Phocaeicola_dorei*, *Phocaeicola_faecalis*, *Phocaeicola_vulgatus*, *Pseudoflavonifractor_capillosus*, and *Raoultella_unclassified* (*P* < 0.05). The MET group showed an increased abundance of *Desulfovibrio_porci*, *Oscillibacter_*sp.*_CU971*, *Phocaeicola_vulgatus*, and *Raoultella_unclassified* in the feces of DKD rats (*P* < 0.05 or 0.01). The MV group showed a significant increase in the abundance of *Allobaculum_unclassified* and *Firmicutes_bacterium_CAG:100_56_8* (*P* < 0.05 or 0.01) and a decrease in the abundance of *Desulfovibrio_porci*, *Oscillibacter_*sp.*_CU971*, *Parablautia_muri*, *Phocaeicola_dorei*, *Phocaeicola_faecalis* (*P* < 0.05 or 0.01). In addition, ML treatment not only modulated the abundance of *Allobaculum_unclassified*, *Desulfovibrio_porci*, *Oscillibacter_*sp.*_CU971*, *Parablautia_muri*, *Phocaeicola_dorei*, *Phocaeicola_faecalis*, *Phocaeicola_vulgatus*, and *Raoultella_unclassified* in the gut of DKD rats but also affected the abundance of *Escherichia_coli* and *Pseudoflavonifractor_capillosus*, which were not influenced by MET or MV (*P* < 0.05 or 0.01).

**FIGURE 9:**
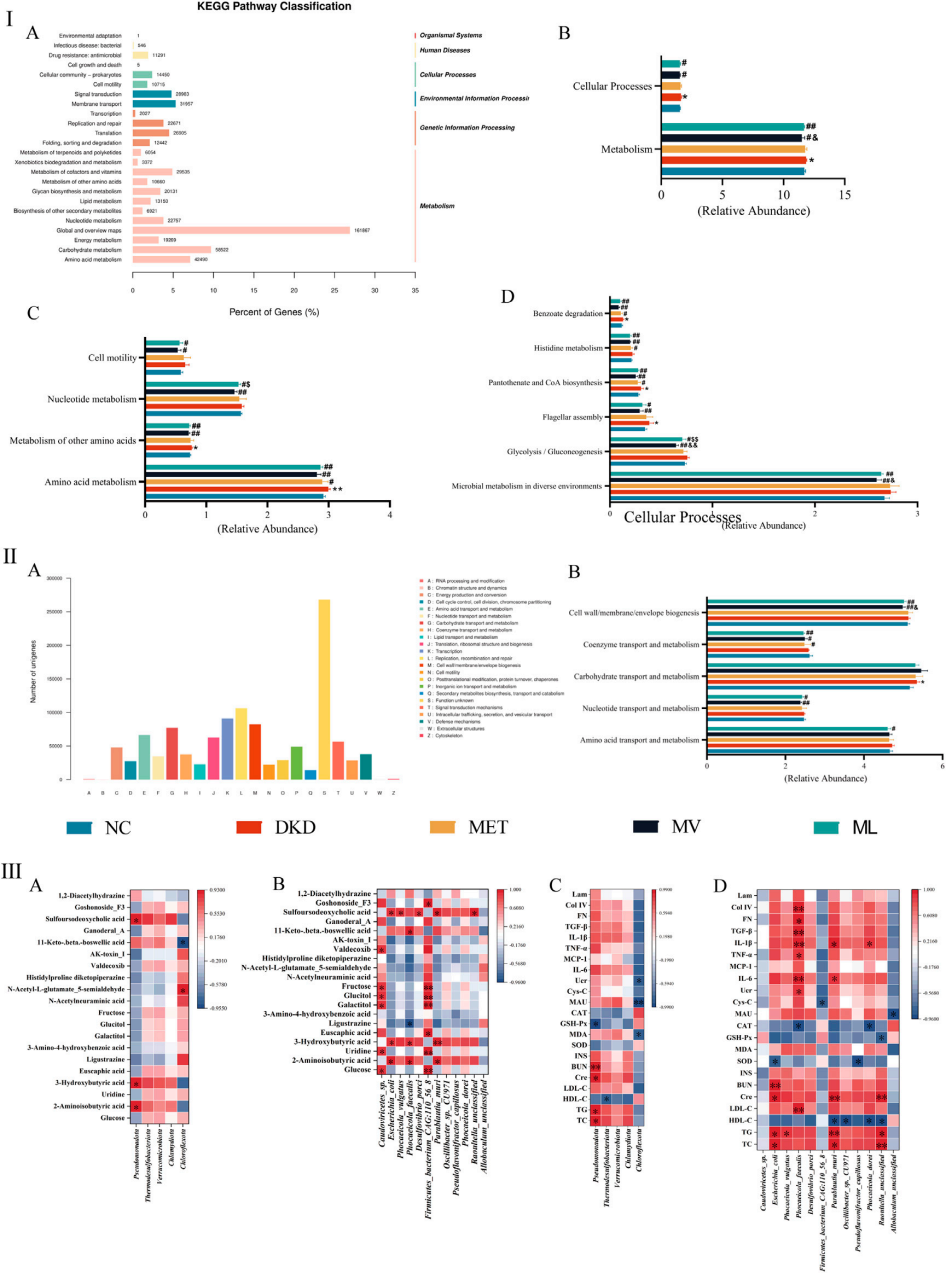
**(Ⅰ)**: Statistical distribution chart of genes associated with Kyoto Encyclopedia of Genes and Genomes (KEGG) metabolic pathways at the secondary classification level **(A)**, and statistical chart of inter-group differences in KEGG functional annotations at the primary **(B)**, secondary **(C)**, and tertiary pathway classification level **(D)**. **(Ⅱ)**: Statistical visualization of functional gene classification based on Evolutionary Genealogy of Genes: Non-supervised Orthologous Groups (eggNOG) **(A)** and comparative statistical visualization of eggNOG-annotated functional divergences across experimental cohorts **(B)**. **(Ⅲ)**: Correlation analysis between differential fecal metabolites and differential gut microbiota at phylum **(A)** or species level **(B)**; Correlation analysis between the biochemical index and gut microbiota at phylum **(C)** or species level **(D)**. BUN, blood urea nitrogen; CAT, catalase; Cre, blood creatinine; Col IV, type IV collagen; Cys-C, cystatin C; FN, fibronectin; GSH-Px, glutathione peroxidase; HDL-C, high-density lipoprotein cholesterol; INS, fasting plasma insulin; IL, interleukin; Lam, laminin; LDL-C, low-density lipoprotein cholesterol; MAU, urinary microalbumin; MCP-1, monocyte chemoattractant protein-1; MDA, malondialdehyde; SOD, superoxide dismutase; TC, total cholesterol; TG, triglyceride; TGF-β, transforming growth factor-β; TNF-α, tumor necrosis factor-α; Ucr, urinary creatinine.

In this study, we annotated non-redundant gene protein sequences against the KEGG database, yielding 24,104 KOs (Figure 9IA). These functions were enriched in 6 level-1 pathways, including metabolism, genetic information processing, environmental information processing, cellular processes, human diseases, and organismal systems, and 24 level-2 and 166 level-3 pathways were identified. A substantial number of functional genes were enriched in metabolic pathways. Among the level-2 pathways, global maps and overviews, carbohydrate metabolism, and amino acid metabolism were notably abundant, indicating a close association between the gut microbiota and metabolism.

Metastats were used for inter-group differential analysis of KEGG pathways, identifying a total of 2 level-1 pathways, 4 level-2 pathways, and 6 level-3 pathways (Figures 9IB–D). At the level-1 pathway level, compared to the DKD group, both the ML and MV groups showed a significant downtrend in the metabolism and cellular processing pathways, with MV having a significantly better impact on metabolism than ML. At the level-2 pathway level, both MV and ML could downregulate amino acid metabolism, other amino acid metabolism, nucleotide metabolism, and cell motility pathways. MV showed a significantly better downregulating effect on nucleotide metabolism than ML, whereas MET only downregulated the amino acid metabolism pathway. At the level-3 pathway level, compared with the NC group, the DKD group showed an upward trend in flagellar components, pantothenate and CoA biosynthesis, and benzoate degradation pathways. MV and ML can downregulate microbial metabolism in different environments, glycolysis/gluconeogenesis, and flagellar component pathways, with MV showing a significantly stronger downregulating effect on glycolysis/gluconeogenesis than ML.

The eggNOG analysis annotated 23 eggNOG level-2 categories, with replication, recombination, and repair having the highest relative quantities, followed by transcription, cell wall/membrane/envelope biogenesis, and carbohydrate transport and metabolism, with similar contents (Figure 9IIA). Employing the Metastats method for inter-group differential analysis of eggNOG functional annotations, five homologous biological functions with significant differences were identified (Figure 9IIB). The results indicated that the DKD group could significantly upregulate carbohydrate transport and metabolism, and the MET group could only significantly reverse the upregulation of coenzyme transport and metabolism functions; both MV and ML groups could significantly downregulate nucleotide transport and metabolism, coenzyme transport and metabolism, and cell wall/membrane/envelope biogenesis functions; the MV group showed a significantly greater downregulating effect on cell wall/membrane/envelope biogenesis than the ML group, while only the ML group significantly downregulated amino acid transport and metabolism functions.

The Pearson correlation analysis results are shown in Figures 9IIIA–D. At the phylum level, *Pseudomonadota* was positively correlated with the metabolites 2-aminoisobutyric acid, 3-hydroxybutyric acid, and sulfoursodeoxycholic acid (*P* < 0.05), whereas *Chloroflexota* was positively correlated with N-acetyl-L-glutamate-5-semialdehyde (*P* < 0.05) and negatively correlated with 11-keto-beta-boswellic acid (*P* < 0.05). In the correlation analysis with biochemical markers, *Pseudomonadota* demonstrated positive correlations with TC, TG, Cre, and BUN (*P* < 0.05 or 0.01), and a negative correlation with GSH-Px (*P* < 0.05). *Thermodesulfobacteriota* showed a negative correlation with HDL-C (*P* < 0.05), *Chloroflexota* was negatively correlated with MDA, MAU, and Ucr (*P* < 0.05 or 0.01). At the species level, *Caudoviricetes* sp. was positively correlated with glucose, uridine, galactitol, glucitol, fructose, and valdecoxib (*P* < 0.05), *Escherichia coli* with 2-aminoisobutyric acid, 3-hydroxybutyric acid, and sulfoursodeoxycholic acid (*P* < 0.05), *Phocaeicola vulgaris* with sulfoursodeoxycholic acid (*P* < 0.05), *Phocaeicola faecalis* with 2-aminoisobutyric acid, 3-hydroxybutyric acid, and 11-keto-beta-boswellic acid (*P* < 0.05), and negatively correlated with ligustrazine (*P* < 0.05), *Desulfovibrio porci* with sulfoursodeoxycholic acid (*P* < 0.05), *Firmicutes bacterium CAG:110_56_8* with glucose, uridine, euscaphic acid, galactitol, glucitol, fructose, and goshonoside F3 (*P* < 0.05 or 0.01), *Parablautia muri* with 2-aminoisobutyric acid, 3-hydroxybutyric acid, and sulfoursodeoxycholic acid (*P* < 0.05 or 0.01), *Raoultella unclassified* with sulfoursodeoxycholic acid (*P* < 0.05). In the correlation analysis with biochemical markers, *E. coli* showed positive correlations with TC, TG, Cre, and BUN (*P* < 0.05 or 0.01), and a negative correlation with SOD (*P* < 0.05); *P. vulgaris* was positively correlated with TG (*P* < 0.05); *P. faecalis* with LDL-C, Ucr, IL-6, TNF-α, IL-1β, TGF-β, FN, and Col IV (*P* < 0.05 or 0.01), and negatively correlated with CAT (*P* < 0.05); *F. bacterium CAG:110_56_8* was negatively correlated with Cys-C (*P* < 0.05); *Parablautia muri* with TC, TG, Cre, IL-6, and IL-1β (*P* < 0.05 or 0.01), and negatively correlated with HDL-C (*P* < 0.05); *Oscillibacter* sp. *CU971* with HDL-C (*P* < 0.05); *Pseudoflavonifractor capillosus* with SOD (*P* < 0.05); *Phocaeicola dorei* with HDL-C and CAT (*P* < 0.05), positively correlated with IL-1β (*P* < 0.05), *R. unclassified* with TC, TG, and Cre (*P* < 0.05 or 0.01), and negatively correlated with HDL-C and GSH-Px (*P* < 0.05); *Allobaculum unclassified* with MAU (*P* < 0.05).

## 4 Discussion

The clinical manifestations of DKD include persistent proteinuria, defined as urinary protein excretion of >300 mg/day or >200 μg/min, confirmed by at least two measurements within 3–6 months, accompanied by a progressive decline in GFR that ultimately leads to ESRD (Thipsawat, 2021). Primary prevention strategies for DKD involve glycemic control, blood pressure management, lipid profile regulation, and lifestyle modifications. MET, widely recognized as the first-line treatment for T2DM, primarily exerts hypoglycemic effects by inhibiting hepatic gluconeogenesis and glycogenolysis to regulate glucose homeostasis (Sanchez-Rangel and Inzucchi, 2017). Studies have indicated that the renin-angiotensin-aldosterone system (RAAS) plays a pivotal role in DKD pathogenesis. RAAS inhibitors dilate both the efferent and afferent glomerular arterioles, reduce intra-glomerular pressure, and decrease proteinuria. Val, a selective angiotensin II receptor blocker, has shown significant efficacy in reducing proteinuria and slowing renal deterioration (Zhou et al., 2024). In the LW formula, the monarch drug Rehmanniae Radix Praeparata nourishes kidney yin, replenishes essence, and augments marrow production; the minister drugs include Dioscoreae Rhizoma, which tonifies qi, nourishes yin, and strengthens the spleen-lung-kidney axis to enhance postnatal biochemical resources; Corni Fructus, which supplements the liver and kidney yin while astringing seminal essence; the assistant drugs consist of Moutan Cortex to clear liver fire, Alismatis Rhizoma to drain dampness and resolve turbidity, and Poria to fortify the spleen and percolate dampness. This formulation embodies the classical “Three Tonifications and Three Drains” therapeutic principle, namely tonify kidney yin (Rehmanniae Radix Praeparata), spleen-pulmonary function (Dioscoreae Rhizoma) and liver-kidney essence (Corni Fructus) and drain pathogenic liver fire (Moutan Cortex), damp-turbidity (Alismatis Rhizoma) and spleen-dampness (Poria). Recent studies have shown that ingredients including phenolic glycosides, furfural derivatives, and polysaccharides in Rehmanniae Radix Praeparata can ameliorate inflammatory responses and oxidative stress and protect pancreatic β-cells (Meng et al., 2021). Dioscoreae Rhizoma polysaccharides can enhance intestinal SCFAs production, improve adipose tissue insulin sensitivity, and activate pyruvate kinase/hexokinase to accelerate glucose utilization (Wang et al., 2023). Morroniside and loganin in Corni Fructus inhibit cellular proliferation, reduce ECM accumulation, and suppress apoptosis and inflammatory cascades to protect against renal injury (Wu et al., 2023). Poria and its triterpenoid components, such as poricoic acid A (PAA), PAB, poricoic acid PZ (PZC), PZD, PZE, PZG, PZH, PZI, PZM, and PZP presented reno-protective effect (Guo et al., 2025). Alisol B 23-acetate (ABA), a triterpenoid isolated from Alismatis rhizoma, can protect renal injury by reducing blood pressure, inhibiting RAS activation, restoring microbial diversity, and repairing intestinal barrier function (Jiang et al., 2025). These polypharmacological profiles from single herbs and/or their active ingredients in LW prescription suggest that LW may exert reno-protective effects via multi-components and multi-targets mechanism. Thus, in this study, we employed Val as a positive control to evaluate LW’s renal protective effects when combined with MET in DKD rats, and based on the clinical doses and relative references, the rat doses of MET, Val, and LW were set at 200 mg/kg, 30 mg/kg, and 6.75 g/kg, respectively. LW has a well-established history of clinical use. This study aimed to validate the therapeutic effects of its specific clinical-equivalent dose in DKD rather than to investigate the dose-response relationship. Thus, only one clinical-equivalent-dose group was included in this study. Our current results demonstrated that the LW + MET and Val + MET combinations significantly outperformed MET monotherapy in improving renal indices, although all treatments showed comparable reductions in blood glucose, body weight, and 24-h urine output.

A hallmark feature of T2DM is IR, and chronic IR can induce functional abnormalities in renal podocytes and tubular cells, including impaired glucose uptake, dysregulated ion transport, and exacerbated apoptosis, ultimately leading to renal dysfunction (Samsu, 2021). The current experimental findings demonstrated that MET, Val + MET, and LW + MET treatments significantly ameliorated abnormal elevations in FBG, HOMA-IR, and insulin levels in DKD rats. The OGTT and ITT results further revealed that LW + MET exhibited superior efficacy in normalizing glucose tolerance compared with Val + MET and MET monotherapy. Whether this synergistic effect is correlated with their pharmacokinetic interactions needs to be further validated and confirmed in future studies.

Chronic hyperglycemia in T2DM triggers vascular endothelial cell damage, which suppresses functional lipoprotein lipase (LPL) activity. This reduction drives pathological increases in TG and decreases in HDL-C levels. Concurrently, diabetic kidney injury exacerbates dyslipidemia; hypoalbuminemia elevates LDL-C, while renal failure specifically amplifies remnant lipoproteins while lowering HDL-C and LDL-C levels (Hirano, 2014). Our study corroborated that MET, Val + MET, and LW + MET effectively reversed plasma abnormalities in TC, TG, and HDL-C levels in DKD rats. Notably, MET alone did not have a significant effect on LDL-C, whereas Val + MET and LW + MET demonstrated robust LDL-C reduction, which is consistent with previous findings on the synergistic lipid-modulating effects of combination therapies (Lin et al., 2016; Meng et al., 2021).

Oxidative stress and inflammation play pivotal roles in renal pathogenesis that form a vicious cycle of cellular injury and fibrotic progression (Liu et al., 2025). Sustained hyperglycemia induces excessive ROS generation, resulting in oxidative damage that subsequently triggers lipid peroxidation and oxidative modification of proteins, ultimately activating apoptotic pathways. Furthermore, oxidative stress stimulates the production of pro-inflammatory growth factors, cytokines, and transcription factors, thereby initiating inflammatory cascades that contribute to organ damage and cellular necrosis (Jha et al., 2016). Emerging evidence indicates that the activation of inflammatory signaling pathways and infiltration of immune cells are pivotal in the pathogenesis of DKD. For example, TNF-α and IL-1β directly regulate monocyte chemoattractant protein-1 (MCP-1) expression, which orchestrates immune-cell activation and recruitment. Simultaneously, IL-6 promotes mesangial cell proliferation, ECM accumulation, and podocyte injury through STAT3-dependent mechanisms, whereas TNF-α exhibits dual cytotoxic effects by exacerbating ROS production, inducing apoptosis, and disrupting the glomerular hemodynamic balance (Chen J. et al., 2022). Notably, in DKD progression, oxidative stress and inflammation reciprocally amplify each other under chronic hyperglycemic conditions, synergistically accelerating the disease pathogenesis. The current study demonstrated that monotherapy with MET exerted limited efficacy in mitigating oxidative stress biomarkers and pro-inflammatory cytokines in rats with DKD. However, synergistic effects were observed for LW + MET and Val + MET combinations. This suggests that LW + MET attenuates DKD progression through dual modulation of oxidative and inflammatory pathways, mechanistically comparable to Val + MET and superior to MET alone.

In clinical practice, UAER and GFR are pivotal biomarkers for monitoring DKD progression. GFR evaluation methodologies included inulin clearance and serum urea, Ucr, and Cys-C measurements. Among these, Cys-C has emerged as the most reliable surrogate marker for GFR owing to its independence from binding protein interference, exclusive glomerular filtration without tubular secretion, and near-complete reabsorption by proximal tubules, rendering its concentration solely reflective of glomerular filtration capacity (Christensson et al., 2004). The current study revealed that the combined administration of MET and either LW or Val exhibited synergistic therapeutic effects in rats with DKD. Specifically, while MET monotherapy moderately reduced MAU levels, its combination with LW or Val not only amplified MAU reduction but also significantly improved Ucr and Cys-C parameters that were unresponsive to MET alone. This underscores the pharmacodynamic synergy between MET and LW/Val in modulating renal filtration and tubular function. Notably, the Val + MET regimen demonstrated superior efficacy in attenuating MAU compared to LW + MET, potentially attributable to Val’s dual angiotensin receptor blockade and hemodynamic regulatory effects that synergize with MET’s metabolic actions. These findings align with earlier mechanistic studies on DKD pathophysiology, where multimodal interventions targeting both oxidative stress and inflammation yielded enhanced renal protection compared to monotherapy.

ECM accumulation in glomerular and tubulointerstitial compartments, along with thickening and hyalinization of the intrarenal vasculature, constitutes another hallmark of DKD (Kanwar et al., 2011). ECM is composed of Col, proteoglycans/glycosaminoglycans, elastin, fibronectin, Lam, and several glycoproteins. Abnormal deposition of these components induces mesangial matrix expansion and thickening of the glomerular basement membrane (GBM), thereby driving microalbuminuria (Mason and Wahab, 2003). Notably, TGF-β1 is a cytokine critically associated with ECM dysregulation. Upon binding to type II serine/threonine kinase receptors, TGF-β1 undergoes phosphorylation-dependent activation of type I receptors, which subsequently interacts with SMAD2/3 to form a heterodimeric complex with SMAD4. Nuclear translocation of this complex facilitates transcriptional regulation of TGF-β1 target genes. Intriguingly, a counter-regulatory loop mediated by SMAD7 operates within the TGF-β1-induced SMAD2/3/4 signaling axis (Leask and Abraham, 2004; Sen et al., 2019). This study demonstrated that combined treatment with MET and either LW or Val significantly ameliorated the renal levels of Col IV, FN, Lam, TGF-β1, and SMAD7 in DKD rats that were not fully responsive to MET monotherapy, which may be attributed to the contribution of active ingredients in Poria and Alismatis rhizome, such as PAA, PAB, PZC, PZD, PZE, PZG, PZH, PZI, PZM, PZP, and ABA presented reno-protective effect (Guo et al., 2025; Jiang et al., 2025). These findings align with those of advanced renal histopathological techniques that quantitatively validate ECM-related structural abnormalities in DKD. This study underscores the therapeutic potential of MET-based combination therapies in interrupting the TGF-β1/SMAD-ECM axis, offering a mechanistic framework for halting the progression of DKD.

To further elucidate the pathogenesis of DKD and the mechanisms of pharmacological interventions, this study employed non-targeted metabolomics based on UHPLC-MS/MS to investigate global changes in endogenous metabolites in fecal samples of DKD rats. Rats with DKD exhibited significant upregulation or downregulation of multiple metabolites compared with normal rats, and the predominant metabolite categories were lipids and lipid-like molecules (51.2%), organic acids and derivatives, organoheterocyclic compounds, and organic oxides. Notably, lipids and lipid-like molecules remained the dominant category across all drug intervention groups, and the correlation analysis revealed strong associations between these differential metabolites and blood lipid parameters. FAs have emerged as the most abundant lipid subclass with regulatory capacities for key transcription factors and biological activities. They play critical roles in modulating gene expression and subsequent biosynthesis of related proteins. For instance, renal FAs are primarily absorbed by proximal tubular cells (Console et al., 2020), while diabetic kidneys display upregulated lipogenic genes and exhibit lipid deposition in both the glomerular and tubular regions (Wang et al., 2005). This study extends the current understanding by integrating metabolomic patterns with established DKD pathways, thereby providing a foundation for developing lipid-focused interventions.

Compared with the DKD group, the endogenous metabolites exhibiting differential changes in the ML group primarily consisted of AAs, peptides, and their analogs. Previous studies have shown that renal dysfunction significantly affects the metabolic processes of AAs, including synthesis, degradation, and filtration. Disruption of AAs metabolic homeostasis might lead to aberrant accumulation of harmful metabolites or activation of related metabolic enzymes, thereby triggering pathways such as oxidative stress, fibrosis, and inflammation during DKD progression (Liu L. et al., 2022). For instance, L-lysine administration in DKD rats has been shown to markedly ameliorate renal injury by modulating metabolic pathways linked to oxidative damage and inflammatory responses (Jozi et al., 2022); clinical studies have indicated a progressive decline in serum levels of BCAAs in DKD patients, which correlates with impaired renal function and metabolic dysregulation (Liu M. et al., 2023).

Pathway enrichment analysis of differential metabolites revealed significant alterations in the metabolic pathways of rats with DKD, including unsaturated FAs biosynthesis, valine/leucine/isoleucine biosynthesis, galactose metabolism, and FAs biosynthesis. MET significantly modulated galactose metabolism, MV-regulated galactose, fructose/mannose, and butyrate metabolism. In contrast, ML demonstrated broader regulatory effects, significantly modulating galactose, butyrate, fructose/mannose, and amino sugar/nucleotide metabolism. Notably, ML treatment reversed the downregulation of galactose metabolism in the DKD rats. Galactose, a critical carbohydrate in cellular metabolism, is predominantly metabolized through three pathways: conversion to glucose-1-phosphate for glycolysis or glycosylation reactions through the Leloir pathway, catabolism via the pentose phosphate shunt through the galactonate pathway, and generation of galactitol excreted through renal clearance via the aldose reductase pathway (Conte et al., 2021). As a vital energy substrate, dysregulation of galactose metabolism is closely associated with metabolic disorders. For instance, partial substitution of dietary glucose with galactose could reduce fasting serum insulin and hepatic triglycerides while attenuating inflammation in female mice (Bouwman et al., 2019). In the butyrate metabolism pathway, 3-hydroxybutyrate levels were markedly elevated in DKD rats, whereas alpha-ketoglutarate (AKG) levels decreased significantly. Elevated 3-hydroxybutyrate levels are associated with diabetic ketosis, and ketogenic diets have been shown to reverse functional renal damage via increased 3-hydroxybutyrate (Poplawski et al., 2011). Conversely, AKG, a key intermediate in the tricarboxylic acid cycle, amino acid biosynthesis, catabolism, and collagen synthesis is associated with renal dysfunction (Guo et al., 2022). Clinical studies have confirmed lower plasma AKG levels in DKD patients than in DM controls (Gordin et al., 2019), corroborating our current findings. Compared with MET, ML uniquely modulated the amino sugar/nucleotide sugar metabolism pathway, significantly upregulating N-acetylneuraminic acid (the predominant member of the sialic acid (SA) family). As an essential component of cell membranes, SA maintains structural integrity and membrane permeability, and serves as a target in oxidative stress responses (Schauer and Kamerling, 2018). This upregulation suggests a protective role of ML-mediated renal preservation. These analyses highlight that the regulation of galactose metabolism, butyrate metabolism, fructose/mannose metabolism, and amino sugar/nucleotide sugar metabolism likely underpins ML’s therapeutic effects of ML on DKD. Mechanistically, ML may mitigate DKD progression by restoring energy homeostasis, modulating oxidative stress, and ameliorating metabolic inflammation.

Gut microbiota and their derived metabolites, such as indoxyl sulfate, p-cresol sulfate, trimethylamine, and trimethylamine N-oxide, and SCFAs, are implicated in the onset and progression of DKD (Zhao et al., 2024). To elucidate the role of intestinal microbiota in ML-mediated DKD intervention, this study employed metagenomic sequencing was used to analyze the structural and compositional alterations of the gut microbiota in DKD rats following ML treatment, along with their correlations with metabolic pathways. Previous studies have demonstrated that patients with DKD exhibit an elevated abundance of *Verrucomicrobiota* phylum, which contributes to increased serum LPS levels and systemic inflammation (Salguero et al., 2019); *Thermodesulfobacteriota* phylum promotes histamine production, influencing gut-mediated anti-inflammatory responses and neurotransmitter activity (Engevik et al., 2024). Although our current data did not show significant enrichment of these two phyla in DKD rats, possibly because of host phenotypic heterogeneity, ML and MV interventions notably reduced their abundance in DKD rats, whereas MET only suppressed *Thermodesulfobacteriota*. This suggests ML’s therapeutic efficacy may partially stem from modulation of these bacterial populations.

A previous study showed that *Allobaculum_unclassified* is associated with vascular endothelial function regulation through the endothelin-1 (ET-1) and vascular endothelial growth factor (VEGF) pathways, exhibiting reduced abundance in DKD rats (Liu T. et al., 2023). Similarly, the current study showed that both MV and ML interventions reversed this decline, potentially ameliorating endothelial dysfunction during DKD progression. In contrast to its reported depletion in T2DM models, *Pseudoflavonifractor_capillosus,* a SCFA-producing bacterium, showed paradoxical enrichment in DKD rats, possibly acting as a conditional pathogen (Yao et al., 2020). ML treatment uniquely normalized its overabundance, suggesting strain-specific roles in the disease context. *Escherichia_coli* showed no significant differences between the NC and DKD groups, a discrepancy possibly linked to host-specific phenotypes (Wang et al., 2022), while ML selectively reduced its abundance, unlike MV or MET, highlighting intervention-specific microbial targeting. Novel bacterial interactions were elucidated, and ML intervention revealed significant modulation of previously unreported taxa in DKD, such as *D. porci*, *Oscillibacter* sp. *CU971*, and *Phocaeicola* species. Although their roles in DKD remain uncharacterized, these bacteria may contribute to LW-mediated renal protection through an unexplored metabolic crosstalk. Functional metagenomic profiling revealed that MV and ML downregulated pathways critical to DKD pathogenesis, including AAs metabolism, nucleotide metabolism, and cellular motility. Notably, eggNOG analysis indicated ML’s unique capacity to regulate AAs transport and metabolism, a finding corroborated by metabolomic correlations between gut microbiota and differential metabolites (e.g., 3-hydroxybutyrate and α-ketoglutarate) linked to biochemical parameters. Pearson correlation analyses further confirmed intricate microbiota-metabolite-disease axis interactions, wherein LW-enhanced microbial homeostasis likely drives metabolite reprogramming to counteract DKD progression. Notably, follow-up validation through animal or cell experiments targeting differential metabolites or microbes may be an effective strategy to further confirm omics and correlation findings.

Collectively, ML exerts multi-targeted effects by reshaping dysbiotic microbiota, restoring protective bacterial consortia, suppressing pathogenic taxa, and recalibrating metabolic flux. These findings underscore the gut-kidney axis as a pivotal therapeutic avenue with LW synergizing MET to amplify microbial metabolic regulation in DKD management.

In summary, this study is the first to investigate the therapeutic efficacy and molecular mechanisms of LW combined with MET in ameliorating DKD-related renal injury, utilizing integrated molecular biology, untargeted metabolomics, and metagenomic approaches. Our findings demonstrate that MET and LW co-treatment not only mitigates glucose-lipid metabolic dysregulation and insulin resistance in DKD rats but also alleviates diabetes-induced renal damage. The underlying mechanisms may involve LW’s multi-targeted effects on (1) attenuating oxidative stress and renal inflammation, (2) modulating TGF-β/SMAD signaling pathway activation, and (3) reprogramming metabolic flux through galactose, fructose/mannose, amino sugar/ribose, and butyrate biosynthesis pathways. Notably, LW synergized with MET to restore gut microbial homeostasis, as evidenced by the significant regulation of *Allobaculum_unclassified*, *Desulfovibrio_porci*, *Escherichia_coli*, *Oscillibacter_*sp.*_CU971*, *Parablautia_muri*, *Pseudoflavonifractor_capillosus*, *Phocaeicola_dorei*, *Phocaeicola_faecalis*, *Phocaeicola_vulgatus*, and *Raoultella_unclassified*. These results provided preclinical evidence supporting MET-LW combination therapy for DKD management, highlighting its dual capacity to rectify host metabolism and gut microbiota dysregulation. The identification of novel microbial targets further underscores the therapeutic potential of microbiome-directed interventions in chronic kidney diseases. Future studies should explore whether these bacterial species play a key role in the pathogenesis of DKD, and the preventive and therapeutic effects of LW and other drugs. This work establishes a framework for integrating microbiota-metabolite networks into DKD therapeutics, offering translational strategies to address both the metabolic and inflammatory etiologies of DKD.

## Data Availability

The metagenomics data supporting the findings of this study are deposited in the Genome Sequence Archive (GSA) in the National Genomics Data Center (NGDC) repository, accession number CRA036084; available at https://ngdc.cncb.ac.cn/gsa/browse/CRA036084. Any other data are available from the corresponding author on reasonable request.
