# Supplementary material for: Liuwei Dihuang pills ameliorate renal injury in experimental type 2 diabetes mellitus rat by regulating host-gut microbiota interaction
Source: Front Pharmacol. 2026 Jan 9;16:1715600. doi: 10.3389/fphar.2025.1715600 (PMC12827525; doi:10.3389/fphar.2025.1715600)
Supplement: Supplementary file 1 [file Supplementaryfile1.docx]

Table S1 List of primers used for RT-PCR

| Gene | Forward primer | Reverse primer |
| --- | --- | --- |
| *β-actin* | CTGAGAGGGAAATCGTGCGTGAC | AGGAAGAGGATGCGGCAGTGG |
| *IL-1β* | TGTTTCCCTCCCTGCCTCTGAC | CGACAATGCTGCCTCGTGACC |
| *TNF-α* | CCGAGATGTGGAACTGGCAGAG | CCACGAGCAGGAATGAGAAGAGG |
| *IL-6* | AGACTTCCAGCCAGTTGCCTTC | GGTCTGTTGTGGGTGGTATCCTC |
| *MCP-1* | AGCCCAGAAACCAGCCAACTC | GCCCAGAAGCGTGACAGAGAC |
| *FN* | GACCAGGTTGATGACACTTCCATTG | TGAGTTCTGTGCTACTGCCTTCTAC |
| *Col IV* | AGATAGCCAAAGCCAAACCCATTC | CACGCAGAGCAGAAGCAAGAAG |
| *Lam* | TGAATGCCTCCACCACAGACC | TCCTCCTGCTGCTCCTTGAAC |
| *TGF-β* | GGACCGCAACAACGCCATC | CACTGCTTCCCGAATGTCTGAC |
| *SMAD7* | CCGCCAGTTCATCAGCAGTTG | CCTCCGCCTGTCCTCTTCTC |
| *SMAD3* | TCAAGAGATACGGATGTTCAAGTGTTC | GTGCCAGCTTCCAGAGATGTATTC |

Table S2 Stability of internal standard (IS) response in quality control (QC) samples

| Internal standard (IS) | Compound | Retention time (s) | Polarity | *m/z* | RSD (%) |
| --- | --- | --- | --- | --- | --- |
| IS1 | Succinic-2¸2¸3¸3-d4 acid | 214.2 | Negative | 121.0445 | 0.97 |
| IS2 | L-Leucine-d3 (methyl-d3) | 160.5 | Negative | 133.1062 | 1.31 |
| IS3 | N-Benzoyl-d5-glycine | 90.8 | Negative | 183.0825 | 2.05 |
| IS4 | Nicotinamide-2¸4¸5¸6-[d4] | 192.2 | Positive | 85.1319 | 0.36 |
| IS5 | Trimethyl-d9-amine N-Oxide | 27.8 | Positive | 127.0801 | 2.66 |
| IS6 | Acetylcholine-d9 chloride (N¸N¸N-trimethyl-d9) | 119.3 | Positive | 155.1736 | 1.88 |

Note: the values of RSD (%) are calculated by the response values of internal standards after repeated detection for five times.

Table S3 Differential metabolites among 5 groups rats

| No | Name | | *m/z* | | RT (s) | | Polarity | | Formula | NC vs DN | | | DN vs MET | | | | | DN vs MV | | | | DN vs ML | | | | |
| --- | --- | --- | --- | --- | --- | --- | --- | --- | --- | --- | --- | --- | --- | --- | --- | --- | --- | --- | --- | --- | --- | --- | --- | --- | --- | --- |
|  |  |  |  |  |  |  |  |  |  | VIP | FC | Trend | VIP | FC | | Trend | | VIP | FC | Trend | | VIP | FC | | Trend | |
| 1 | Glucose | | 179.06 | | 169 | | NEG | | C_6_H_12_O_6_ | 1.33 | 0.04 | ↓ |  |  | |  | | 1.73 | 2.52 | ↑↑ | | 1.14 | 2.11 | | ↑ | |
| 2 | Glycocholic acid | | 466.32 | | 148.5 | | POS | | C_26_H_43_NO_6_ | 1.47 | 6.41 | ↑↑↑ | 1.62 | 0.46 | | ↓↓ | |  |  |  | |  |  | |  | |
| 3 | Tauro-γ -muricholic acid | | 514.29 | | 82.1 | | NEG | | C_26_H_45_NO_7_S | 1.09 | 0.07 | ↓ | 1.91 | 2.85 | | ↑ | |  |  |  | |  |  | |  | |
| 4 | 2-Aminoisobutyric acid | | 104.07 | | 196.8 | | POS | | C_4_H_9_NO_2_ | 1.23 | 2.28 | ↑↑ |  |  | |  | |  |  |  | | 1.54 | 0.39 | | ↓↓ | |
| 5 | Uridine | | 243.06 | | 68.9 | | NEG | | C_9_H_12_N_2_O_6_ | 1.45 | 0.00 | ↓ | 1.65 | 4.32 | | ↑ | | 2.01 | 7.18 | ↑ | | 1.54 | 3.72 | | ↑ | |
| 6 | 2-Methylbutyrylglycine | | 158.08 | | 87.9 | | NEG | | C_7_H_13_NO_3_ | 1.04 | 0.16 | ↓ |  |  | |  | | 1.39 | 2.62 | ↑ | |  |  | |  | |
| 7 | 3-Hydroxybutyric acid | | 103.04 | | 120.8 | | NEG | | C_4_H_8_O_3_ | 1.03 | 5.53 | ↑ | 1.47 | 0.30 | | ↓ | | 1.67 | 0.12 | ↓ | | 1.70 | 0.05 | | ↑↑ | |
| 8 | Isobutyrylglycine | | 144.07 | | 107.8 | | NEG | | C_6_H_11_NO_3_ | 1.16 | 0.15 | ↓ |  |  | |  | | 1.73 | 4.07 | ↑↑ | |  |  | |  | |
| 9 | Tauroursodeoxycholic acid | | 498.29 | | 38.7 | | NEG | | C_26_H_45_NO_6_S | 1.04 | 0.11 | ↓ | 1.68 | 2.34 | | ↑ | |  |  |  | |  |  | |  | |
| 10 | 3-Dehydroquinic acid | | 189.04 | | 162 | | NEG | | C_7_H_10_O_6_ | 1.41 | 0.02 | ↓ |  |  | |  | | 1.72 | 2.23 | ↑↑ | |  |  | |  | |
| 11 | Cytosine | | 112.05 | | 101.3 | | POS | | C_4_H_5_N_3_O | 1.28 | 4.94 | ↑↑↑ |  |  | |  | | 1.75 | 0.42 | ↓↓ | |  |  | |  | |
| 12 | Euscaphic acid | | 487.34 | | 31.4 | | NEG | | C_30_H_48_O_5_ | 1.31 | 0.08 | ↓ |  |  | |  | |  |  |  | | 1.52 | 2.17 | | ↑↑ | |
| 13 | Ligustrazine | | 137.11 | | 27.3 | | POS | | C_8_H_12_N_2_ | 1.33 | 0.46 | ↓↓ |  |  | |  | |  |  |  | | 1.57 | 2.04 | | ↑↑ | |
| 14 | 3-Amino-4-hydroxybenzoic acid | | 154.05 | | 199 | | POS | | C_7_H_7_NO_3_ | 1.40 | 0.48 | ↓↓↓ |  |  | |  | |  |  |  | | 1.84 | 3.78 | | ↑↑↑ | |
| 15 | Glucitol | | 181.07 | | 158 | | NEG | | C_6_H_14_O_6_ | 1.35 | 0.04 | ↓ | 1.96 | 5.07 | | ↑ | | 1.76 | 5.77 | ↑ | | 1.47 | 2.61 | | ↑↑ | |
| 16 | Taurodeoxycholic acid | | 498.29 | | 38.7 | | NEG | | C_26_H_45_NO_6_S | 1.04 | 0.11 | ↓ | 1.68 | 2.34 | | ↑ | |  |  |  | |  |  | |  | |
| 17 | Galactitol | | 181.07 | | 158 | | NEG | | C_6_H_14_O_6_ | 1.35 | 0.04 | ↓ | 1.96 | 5.07 | | ↑ | | 1.76 | 5.77 | ↑ | | 1.47 | 2.61 | | ↑↑ | |
| 18 | Fructose | | 179.06 | | 169 | | NEG | | C_6_H_12_O_6_ | 1.33 | 0.04 | ↓ |  |  | |  | | 1.73 | 2.52 | ↑↑ | | 1.14 | 2.11 | | ↑ | |
| 19 | N-Acetylneuraminic acid | | 308.10 | | 168.5 | | NEG | | C_11_H_19_NO_9_ | 1.33 | 0.04 | ↓ |  |  | |  | |  |  |  | | 1.38 | 2.51 | | ↑ | |
| 20 | Deoxyribose | | 133.05 | | 155.8 | | NEG | | C_5_H_10_O_4_ | 1.46 | 0.01 | ↓ |  |  | |  | |  |  |  | | 1.92 | 3.87 | | ↑↑↑ | |
| 21 | Isovalerylalanine | | 174.11 | | 169.2 | | POS | | C_8_H_15_NO_3_ | 1.10 | 2.53 | ↑↑ | 1.88 | 0.40 | | ↓↓ | | 1.74 | 0.35 | ↓↓ | |  |  | |  | |
| 22 | (3R)-3,4-Dihydroxy-3-(hydroxymethyl)butanenitrile_4-glucoside | | 294.12 | | 176.6 | | POS | | C_11_H_19_NO_8_ | 1.01 | 2.22 | ↑ | 1.73 | 0.42 | | ↓ | | 1.92 | 0.30 | ↓↓ | |  |  | |  | |
| 23 | N-Acetyl-L-glutamate-5-semialdehyde | | 174.08 | | 52.7 | | POS | | C_7_H_11_NO_4_ | 1.17 | 0.29 | ↓ |  |  | |  | |  |  |  | | 1.31 | 2.03 | | ↑↑ | |
| 24 | 16α,17β-Estriol_16-(β-D-glucuronide) | 463.20 | | 168.5 | | NEG | | C_24_H_32_O_9_ | | 1.42 | 0.02 | ↓ | 1.65 | | 3.99 | | ↑ |  |  | |  |  | |  | |  |
| 25 | Histidylproline diketopiperazine | 233.10 | | 72.4 | | POS | | C_12_H_16_N_4_O_2_ | | 1.17 | 0.35 | ↓↓ |  | |  | |  |  |  | |  | 1.30 | | 3.32 | | ↑ |
| 26 | Valdecoxib | 313.07 | | 59.8 | | NEG | | C_16_H_14_N_2_O_3_S | | 1.34 | 0.03 | ↓ |  | |  | |  |  |  | |  | 1.56 | | 2.66 | | ↑↑ |
| 27 | 3β-3-Hydroxy-18-lupen-21-one | 441.37 | | 17 | | POS | | C_30_H_48_O_2_ | | 1.42 | 0.11 | ↓ | 1.72 | | 2.06 | | ↑ |  |  | |  | 1.51 | | 2.26 | | ↑ |
| 28 | AK-toxin_I | 414.20 | | 295.9 | | POS | | C_23_H_27_NO_6_ | | 1.12 | 0.29 | ↓↓ |  | |  | |  | 1.39 | 2.04 | | ↑ | 1.21 | | 2.36 | | ↑ |
| 29 | 11-Keto-.beta.-boswellic acid | 471.35 | | 130.3 | | POS | | C_30_H_46_O_4_ | | 1.10 | 4.64 | ↑↑ |  | |  | |  | 1.44 | 0.25 | | ↓↓ |  | |  | |  |
| 30 | 2,9-Bis(3-methyl-2E-pentenoyl)-2b,9a-dihydroxy-4Z,10(14)-oplopadien-3-one | 443.28 | | 50.7 | | POS | | C_27_H_38_O_5_ | | 1.36 | 0.26 | ↓↓↓ |  | |  | |  |  |  | |  | 1.41 | | 2.11 | | ↑↑ |
| 31 | trans-9,11-octadecadienoic_acid | 281.25 | | 17.2 | | POS | | C_18_H_32_O_2_ | | 1.27 | 2.42 | ↑↑ |  | |  | |  | 1.80 | 0.40 | | ↓↓ |  | |  | |  |
| 32 | Corchorusoside_A | 699.36 | | 121.6 | | POS | | C_35_H_54_O_14_ | | 1.30 | 0.03 | ↓ |  | |  | |  | 1.42 | 4.89 | | ↑ |  | |  | |  |
| 33 | 4--2,3,7-trihydroxy-10,13-dimethylhexadecahydro-1H-cyclopenta phenanthren-17-yl)pentanoic acid | 391.28 | | 40.7 | | POS | | C_24_H_40_O_5_ | | 1.25 | 3.70 | ↑↑↑ |  | |  | |  | 2.11 | 0.20 | | ↓↓↓ |  | |  | |  |
| 34 | Ganoderal_A | 437.34 | | 18.6 | | POS | | C_30_H_44_O_2_ | | 1.34 | 0.21 | ↓ |  | |  | |  |  |  | |  | 1.39 | | 2.20 | | ↑ |
| 35 | Sudca Sulfoursodeoxycholic acid | 471.24 | | 178.4 | | NEG | | C_24_H_40_O_7_S | | 1.01 | 12.50 | ↑ | 2.19 | | 0.02 | | ↓ | 1.68 | 0.04 | | ↓ | 1.33 | | 0.07 | | ↓ |
| 36 | Goshonoside_F3 | 645.35 | | 184 | | POS | | C_32_H_52_O_13_ | | 1.37 | 0.09 | ↓ |  | |  | |  |  |  | |  | 1.24 | | 2.03 | | ↑ |
| 37 | 1,2-Diacetylhydrazine | 117.07 | | 214.4 | | POS | | C_4_H_8_N_2_O_2_ | | 1.31 | 2.07 | ↑↑↑ |  | |  | |  |  |  | |  | 1.72 | | 0.46 | | ↓↓↓ |
| 38 | 4-3,4,7,12-tetrahydroxy-10,13-dimethylhexadecahydro-1H-cyclopenta[a]phenanthren-17-yl)pentanoic acid | 407.28 | | 48 | | POS | | C_24_H_40_O_6_ | | 1.05 | 2.51 | ↑ | 2.14 | | 0.49 | | ↓ | 1.67 | 0.44 | | ↓↓ |  | |  | |  |
| 39 | Methyl-acrylate-divinylbenzene,completely-hydrolyzed,copolymer | 399.12 | | 194 | | POS | | C_18_H_18_N_6_O_3_S | | 1.31 | 8.29 | ↑ | 2.08 | | 0.21 | | ↓ | 1.20 | 0.39 | | ↓ |  | |  | |  |
| 40 | SM(d18:1/14:0) | 675.55 | | 25.2 | | POS | | C_37_H_75_N_2_O_6_P | | 1.28 | 10.83 | ↑ |  | |  | |  | 1.85 | 0.16 | | ↓ |  | |  | |  |
| 41 | Rigin | 457.25 | | 249.5 | | POS | | C_18_H_32_N_8_O_6_ | | 1.21 | 4.21 | ↑ | 2.31 | | 0.11 | | ↓↓ | 1.77 | 0.21 | | ↓↓ |  | |  | |  |
| 42 | Prehumulinic acid | 281.18 | | 157.3 | | POS | | C_16_H_24_O_4_ | | 1.26 | 0.21 | ↓ | 2.45 | | 148.1 | | ↑↑ | 2.08 | 146.2 | | ↑ |  | |  | |  |

Table S4 Differential metabolites presented in MET vs MV, MET vs ML, and MV vs ML

| No. | Name | m/z | RT (s) | Polarity | Formula | MET vs MV | | | MET vs ML | | | MV vs ML | | |
| --- | --- | --- | --- | --- | --- | --- | --- | --- | --- | --- | --- | --- | --- | --- |
|  |  |  |  |  |  | VIP | FC | P | VIP | FC | P | VIP | FC | P |
| 1 | Glucose | 179.06 | 169 | NEG | C_6_H_12_O_6_ |  |  |  |  |  |  | 0.55 | 0.84 | 0.40 |
| 2 | Uridine | 243.06 | 68.9 | NEG | C_9_H_12_N_2_O_6_ | 1.19 | 1.66 | 0.20 | 0.07 | 0.86 | 0.71 | 1.09 | 0.52 | 0.10 |
| 3 | 3-Hydroxybutyric acid | 103.04 | 120.8 | NEG | C_4_H_8_O_3_ | 1.25 | 0.41 | 0.17 | 1.46 | 0.18 | 0.06 | 0.55 | 0.43 | 0.32 |
| 4 | Glucitol | 181.07 | 158 | NEG | C_6_H_14_O_6_ | 0.16 | 1.14 | 0.73 | 1.20 | 0.51 | 0.10 | 1.00 | 0.45 | 0.10 |
| 5 | Galactitol | 181.07 | 158 | NEG | C_6_H_14_O_6_ | 0.16 | 1.14 | 0.73 | 1.20 | 0.51 | 0.10 | 1.00 | 0.45 | 0.10 |
| 6 | Fructose | 179.06 | 169 | NEG | C_6_H_12_O_6_ |  |  |  |  |  |  | 0.55 | 0.84 | 0.40 |
| 7 | Isovalerylalanine | 174.11 | 169.2 | POS | C_8_H_15_NO_3_ | 0.47 | 0.89 | 0.56 |  |  |  |  |  |  |
| 8 | (3R)-3,4-Dihydroxy-3-(hydroxymethyl) butanenitrile_4-glucoside | 294.12 | 176.6 | POS | C_11_H_19_NO_8_ | 0.33 | 0.70 | 0.33 |  |  |  |  |  |  |
| 9 | 3β-3-Hydroxy-18-lupen-21-one | 441.37 | 17 | POS | C_30_H_48_O_2_ |  |  |  | 0.33 | 1.10 | 0.71 |  |  |  |
| 10 | AK-toxin_I | 414.20 | 295.9 | POS | C_23_H_27_NO_6_ |  |  |  |  |  |  | 0.31 | 1.16 | 0.58 |
| 11 | Sulfoursodeoxycholic acid | 471.24 | 178.4 | NEG | C_24_H_40_O_7_S | 1.89 | 2.48 | 0.009 | 1.91 | 3.89 | 0.00 | 1.11 | 1.57 | 0.02 |
| 12 | 4-3,4,7,12-tetrahydroxy-10,13-dimethylhexadecahydro-1H-cyclopenta[a]phenanthren-17-yl) pentanoic acid | 407.28 | 48 | POS | C_24_H_40_O_6_ | 0.75 | 0.90 | 0.47 |  |  |  |  |  |  |
| 13 | Methyl-acrylate-divinylbenzene,completely-hydrolyzed,copolymer | 399.12 | 194 | POS | C_18_H_18_N_6_O_3_S | 0.81 | 1.83 | 0.19 |  |  |  |  |  |  |
| 14 | Rigin | 457.25 | 249.5 | POS | C_18_H_32_N_8_O_6_ | 1.31 | 1.91 | 0.13 |  |  |  |  |  |  |
| 15 | Prehumulinic acid | 281.18 | 157.3 | POS | C_16_H_24_O_4_ | 0.33 | 0.99 | 0.97 |  |  |  |  |  |  |

Table S5 Quality control statistical summary of metagenomic sequencing data

| Group | Clean data read | Valid data read | Q20 (%) | Q30 (%) | GC (%) |
| --- | --- | --- | --- | --- | --- |
| NC | 37031697.67±2207692.98 | 27929875±2264045.82 | 99.09±0.03 | 96.92±0.1 | 46.14±0.71 |
| DN | 35999851.33±2502978.20 | 26168093.67±9446980.22 | 99.01±0.08 | 96.73±0.20 | 48.32±2.0 |
| MET | 36810926±2561959.99 | 33346355.67±2849744.19 | 99.08±0.06 | 96.90±0.17 | 47.67±3.45 |
| MV | 35726527.67±1399847.71 | 31427484.67±1904080.75 | 99.04±0.05 | 96.80±0.13 | 50.45±2.74 |
| ML | 35780817.33±1848499.02 | 30531292.67±2113169.51 | 99.04±0.05 | 96.81±0.19 | 49.10±2.88 |

Note: Q20 (%), the proportion of bases in filtered sequencing data with a Phred quality score ≥20; Q30 (%), the percentage of bases in qualified sequencing data meeting a Phred quality score ≥30; GC (%), the ratio of guanine (G) and cytosine (C) bases within the filtered sequencing dataset.

Table S6 Metagenomic assembly statistics

| Group | Contig Num | Largest contig | Total length (bp) | N50 (bp) |
| --- | --- | --- | --- | --- |
| NC | 123966.17±17231.29 | 480417.33±79821.98 | 188319138.3±11973158.555 | 2362.17±771.63 |
| DN | 108025.17±25288.38 | 429131.67±167376.42 | 160910803±45101366.89 | 2093.67±564.22 |
| MET | 79495±13958 | 522862±231278.29 | 137049652.3±30739945.8 | 3436±1018.06 |
| MV | 77667.67±20768.66 | 449664.5±34041.64 | 117095182.7±30672293.71 | 2162.33±437.68 |
| ML | 66888.83±9380.94 | 606492.33±207293.08 | 114866973.2±26452045.88 | 3081.33±925.52 |

Note: Contig Num., Number of assembled contigs; Largest contig, Nucleotide count of the largest contig; Total length, total assembled length of contigs; N50 is calculated by sorting all contigs in descending order of their lengths and sequentially summing their sizes until the cumulative total reaches half of the genome assembly length. The length of the contig at this critical summation point defines the N50 value.


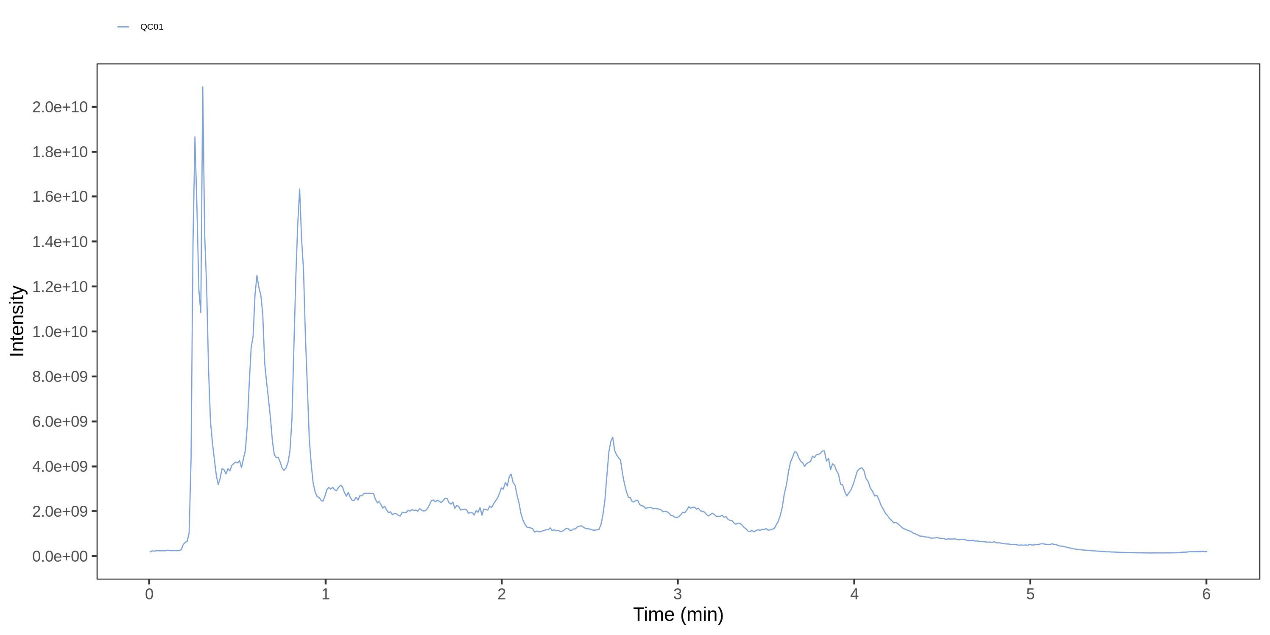


**Fig. S1** Total ion chromatogram (TIC) of quality control (QC) samples analyzed in positive ion mode using ultra-high performance liquid chromatography-tandem mass spectrometry (UHPLC-MS/MS).


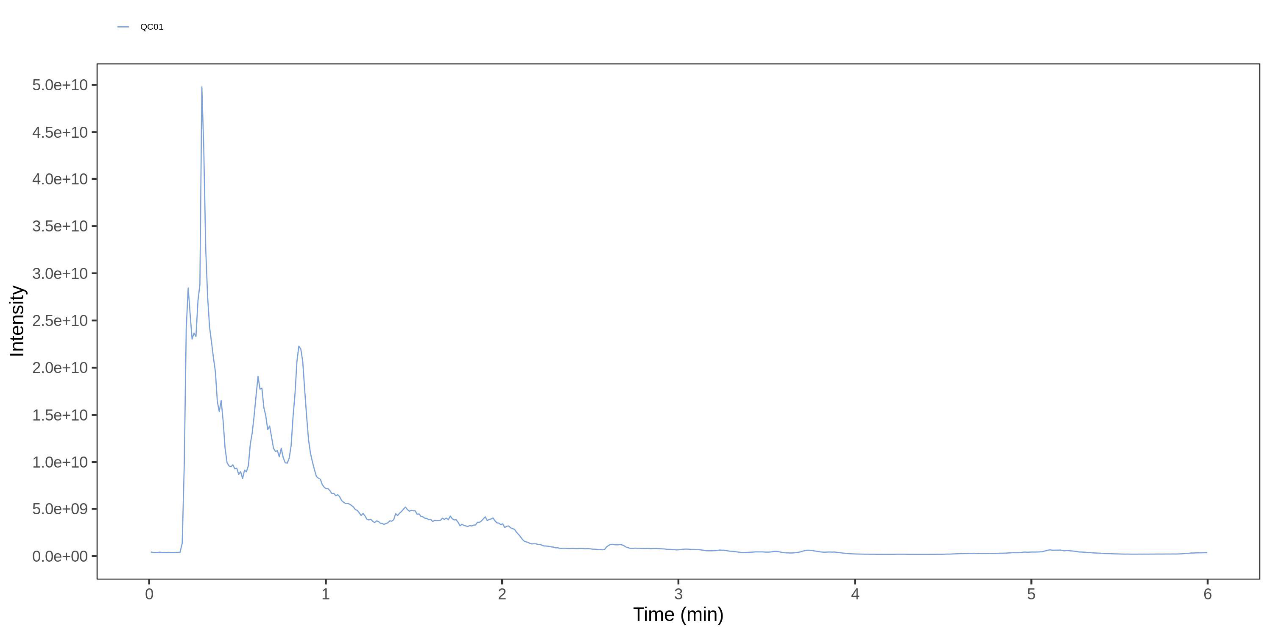


**Fig. S2** Total ion chromatogram (TIC) of quality control (QC) samples analyzed in negative ion mode using ultra-high performance liquid chromatography-tandem mass spectrometry (UHPLC-MS/MS).


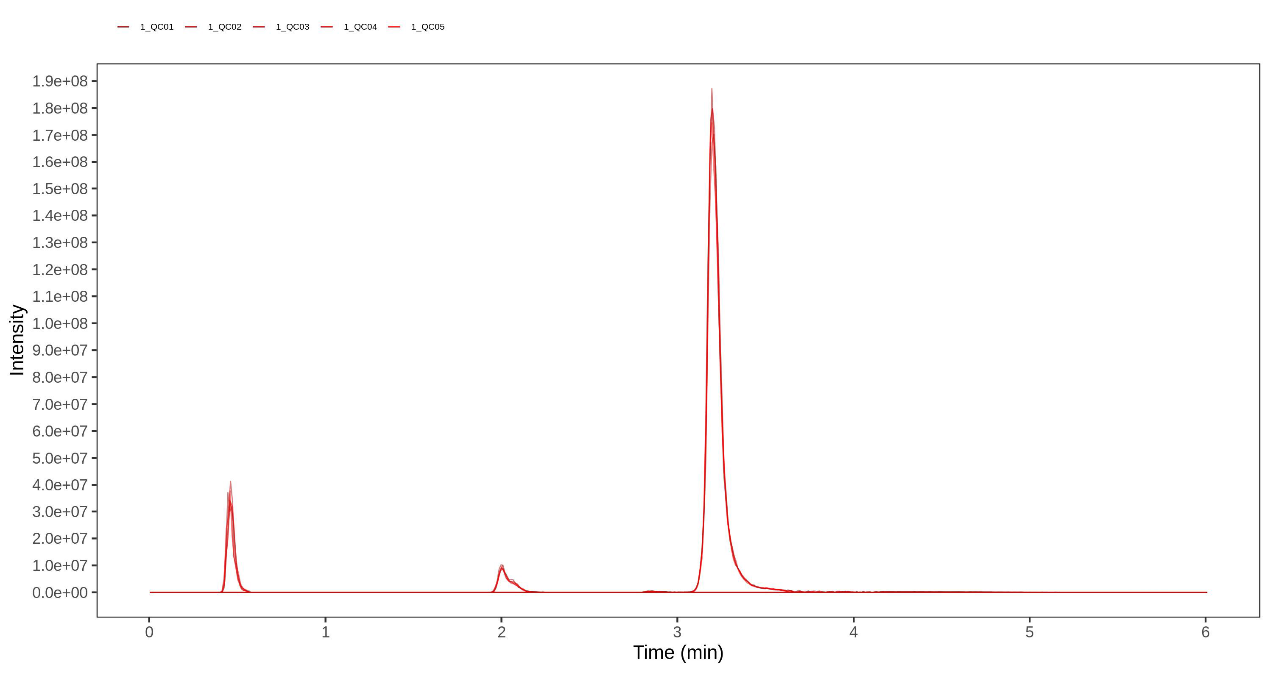


**Fig. S3** Extracted ion chromatograms (EICs) of internal standards in quality control (QC) samples analyzed in positive ion mode using ultra-high performance liquid chromatography-tandem mass spectrometry (UHPLC-MS/MS).


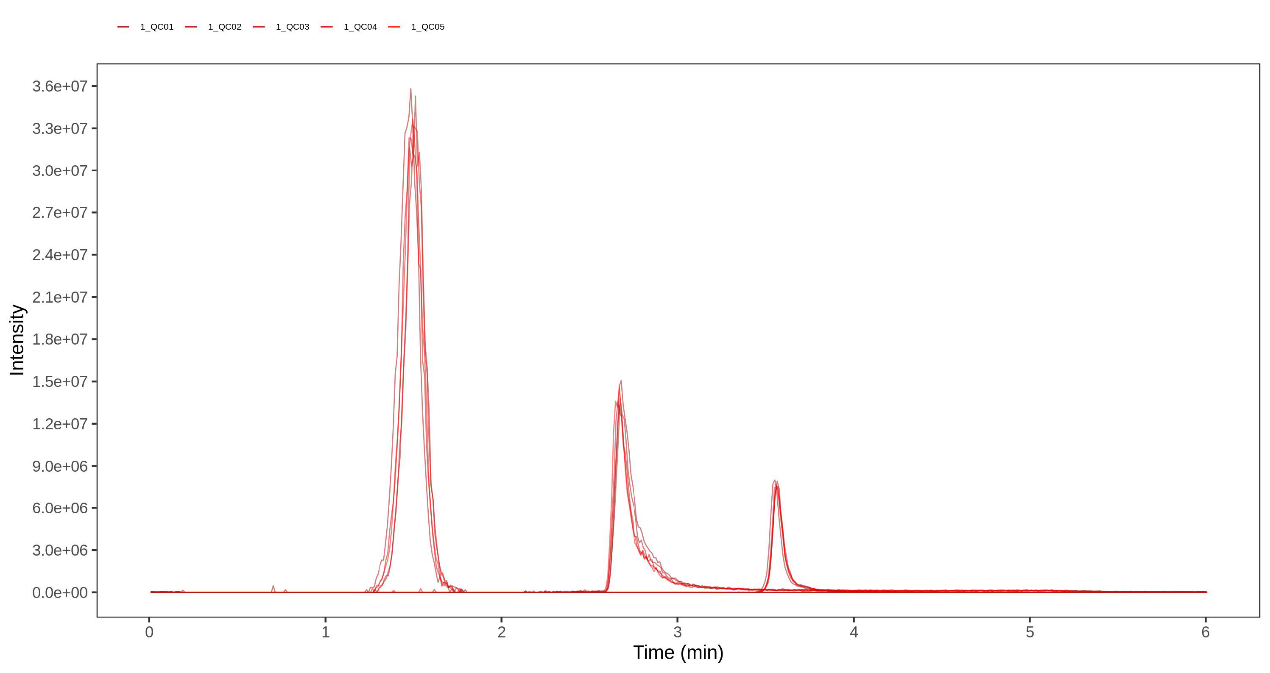


**Fig. S4** Extracted ion chromatograms (EICs) of internal standards in quality control (QC) samples analyzed in negative ion mode using ultra-high performance liquid chromatography-tandem mass spectrometry (UHPLC-MS/MS).


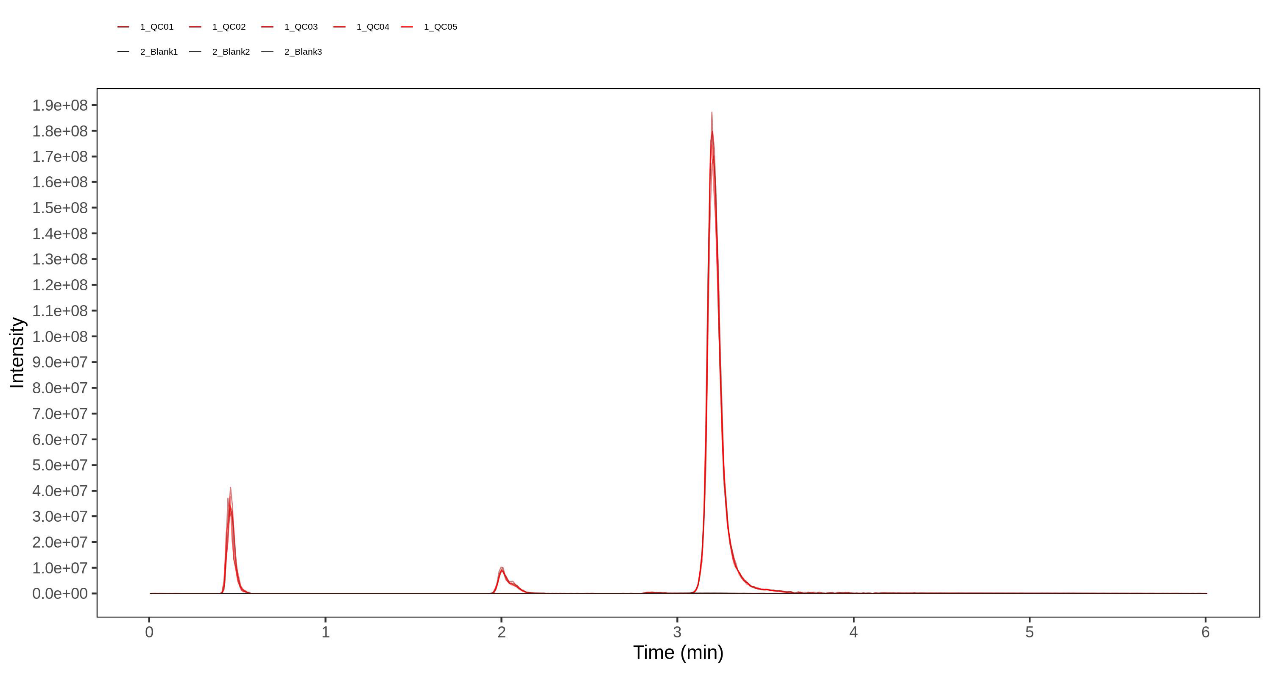
**Fig. S5** Extracted ion chromatograms (EICs) of internal standards in quality control (QC) samples and blank samples analyzed in positive ion mode using ultra-high performance liquid chromatography-tandem mass spectrometry (UHPLC-MS/MS).


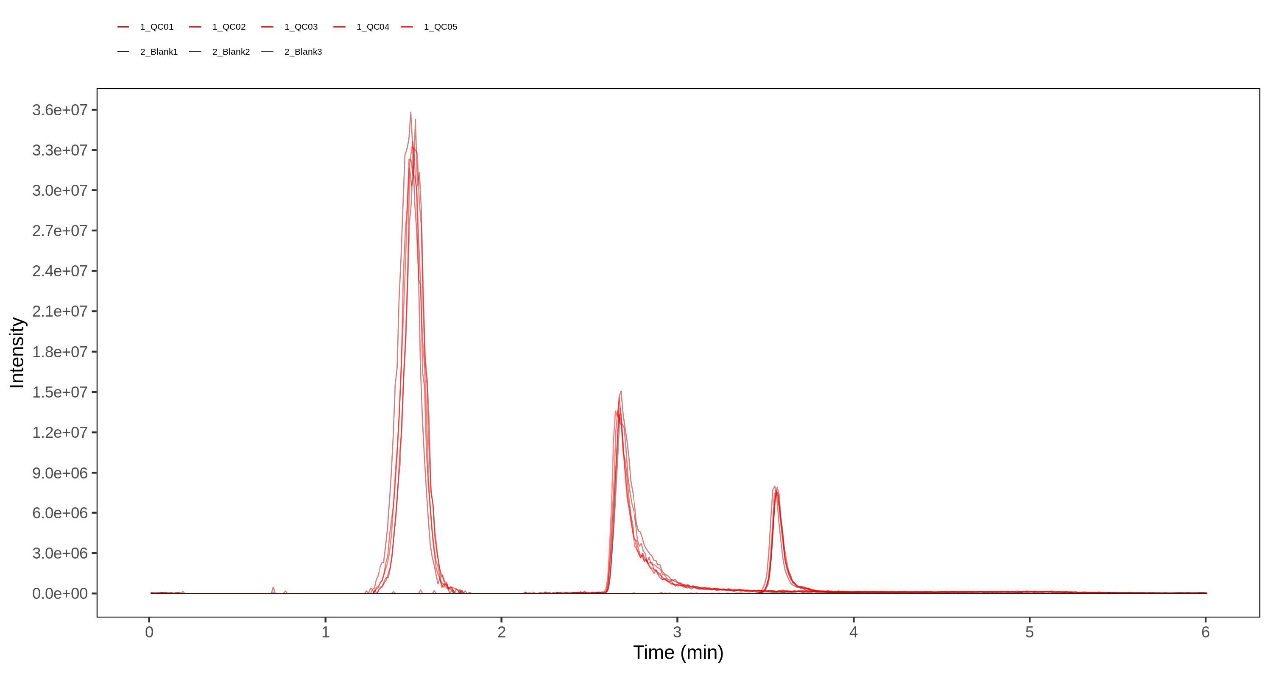


**Fig. S6** Extracted ion chromatograms (EICs) of internal standards in quality control (QC) samples and blank samples analyzed in negative ion mode using ultra-high performance liquid chromatography-tandem mass spectrometry (UHPLC-MS/MS).


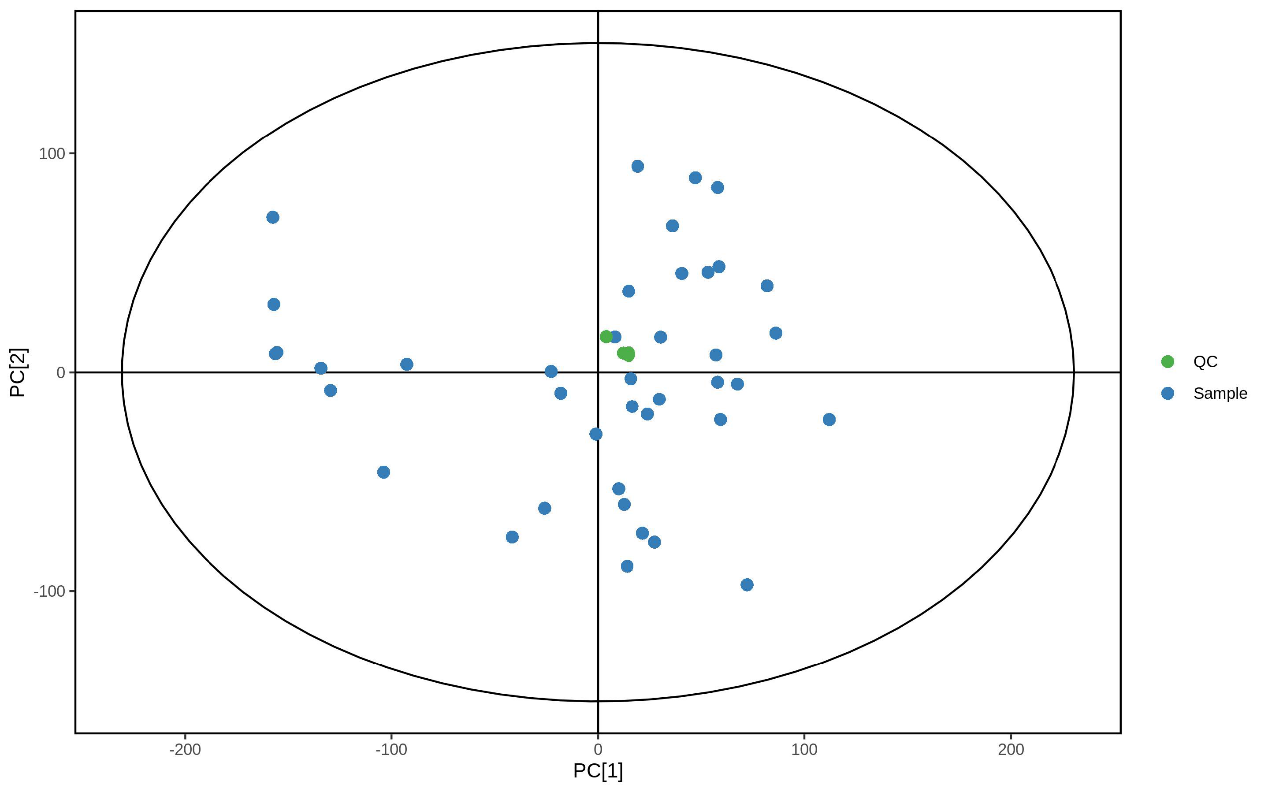


**Fig. S7** Principal component analysis (PCA) score plot. Green dots represent the quality control samples. Blue dots represent the real fecal samples.


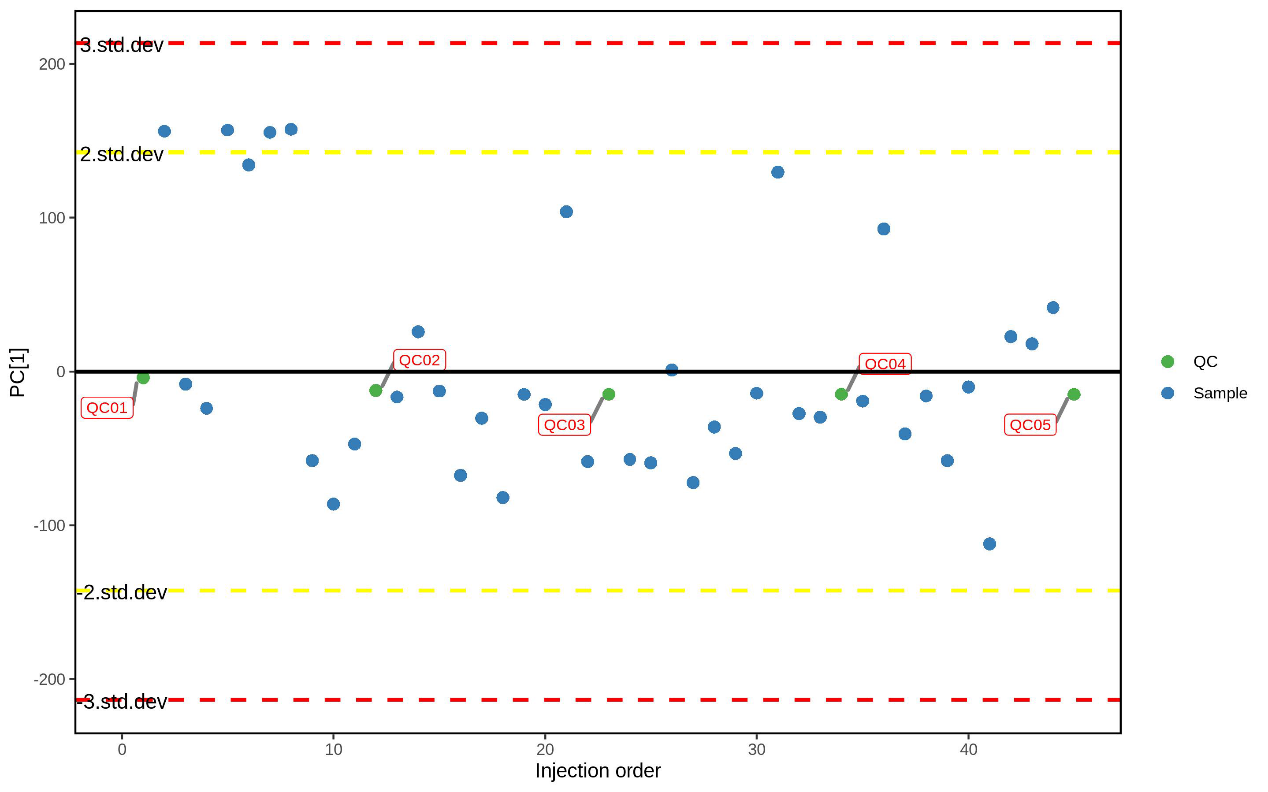


**Fig. S8** One-dimensional PCA-X distribution plot. Green dots represent the quality control samples. Blue dots represent the real fecal samples.


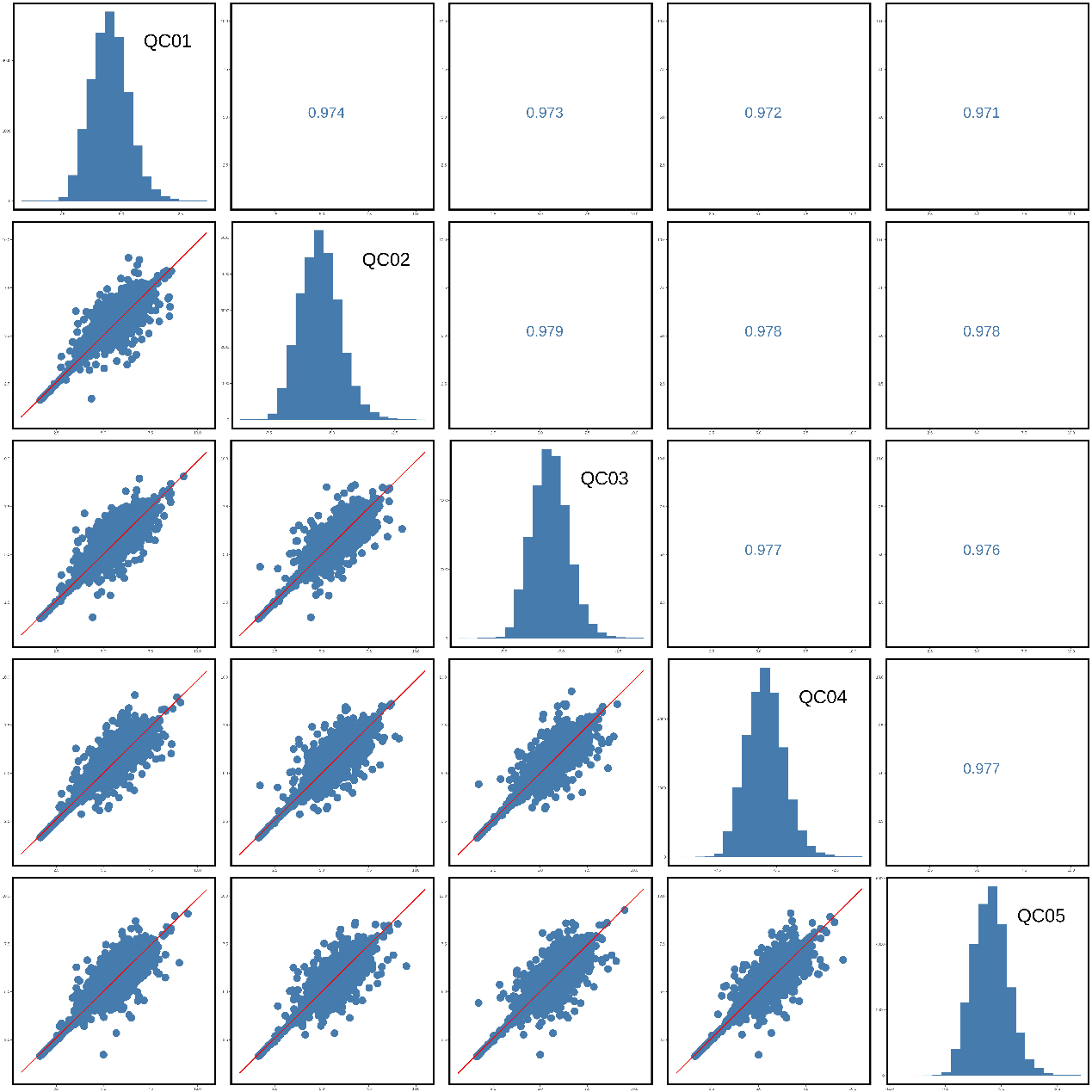


**Fig. S9** Inter-sample correlation analysis of quality control (QC) samples.


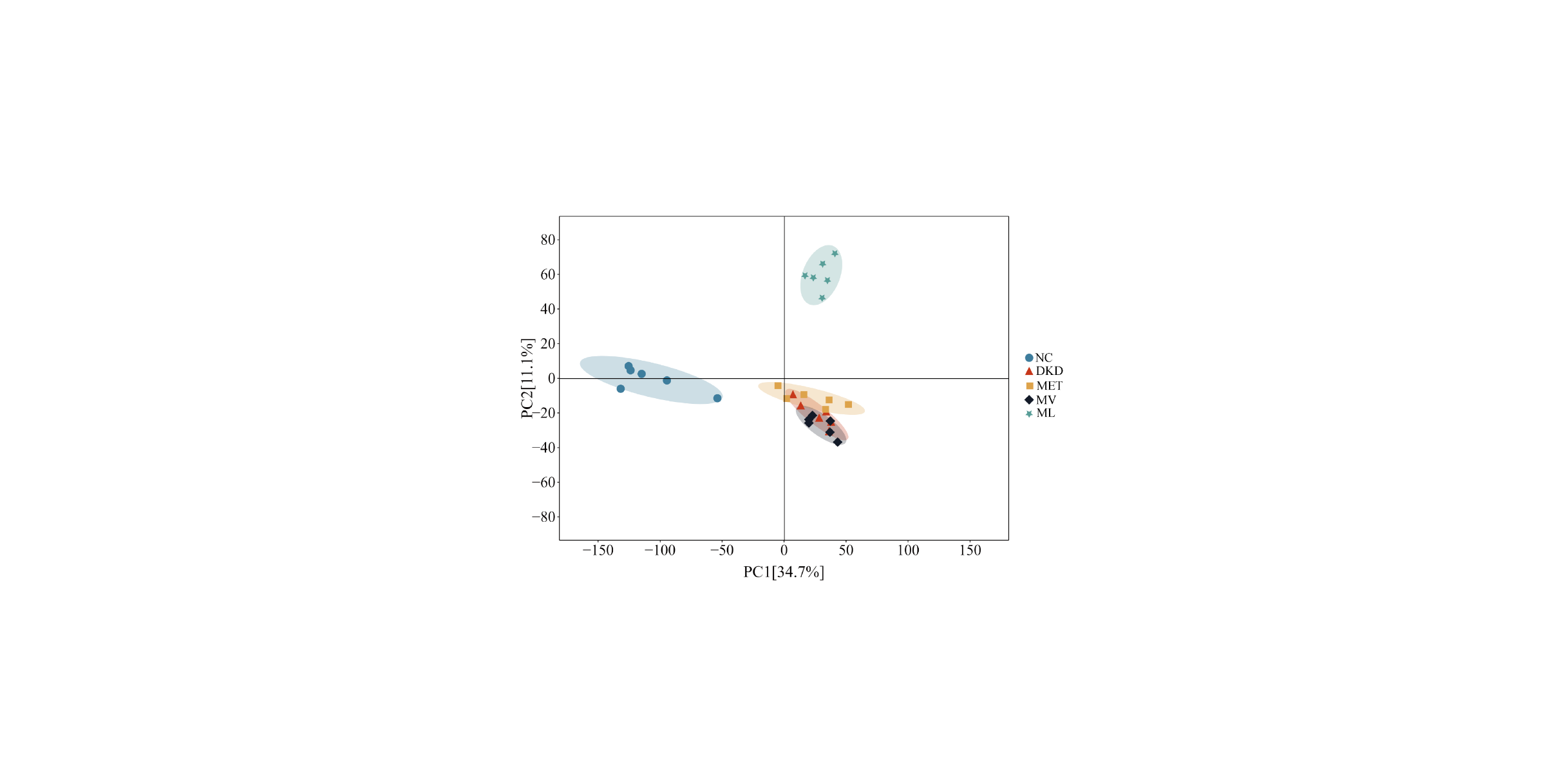


F**ig. S10** Principal component analysis (PCA) plot across five experimental groups of rat fecal samples. NC, DKD, MET, MV, and ML represent the normal control group, diabetic kidney disease group, DKD group treated with metformin of 200 mg/kg, DKD group treated with metformin of 200 mg/kg and valsartan of 30 mg/kg, and DKD group treated with metformin of 20 mg/kg and Liuwei Dihuang pills of 6.75 g/kg, respectively.
